# Antifungal Potential of Green Synthesized Magnetite Nanoparticles Black Coffee–Magnetite Nanoparticles Against Wilt Infection by Ameliorating Enzymatic Activity and Gene Expression in *Solanum lycopersicum* L.

**DOI:** 10.3389/fmicb.2022.754292

**Published:** 2022-03-03

**Authors:** Hina Ashraf, Tanzeela Batool, Tehmina Anjum, Aqsa Illyas, Guihua Li, Shahzad Naseem, Saira Riaz

**Affiliations:** ^1^Guangdong Key Laboratory for New Technology Research of Vegetables, Vegetable Research Institute, Guangdong Academy of Agricultural Sciences, Guangzhou, China; ^2^Centre of Excellence in Solid State Physics, University of the Punjab, Lahore, Pakistan; ^3^Department of Plant Pathology, Faculty of Agricultural sciences, University of the Punjab, Lahore, Pakistan

**Keywords:** Fe_3_O_4_ NPs, antifungal, extracts, tomato, spinach, defense enzymes, genes

## Abstract

Tomato plants are prone to various biotic and abiotic stresses. Fusarium wilt is one of the most devasting diseases of tomatoes caused by *Fusarium oxysporum* f. sp. *lycopersici*, causing high yield and economic losses annually. Magnetite nanoparticles (Fe_3_O_4_ NPs) are one of the potent candidates to inhibit fungal infection by improving plant growth parameters. Spinach has been used as a starting material to synthesize green-synthesized iron oxide nanoparticles (IONPs). Various extracts, i.e., pomegranate juice, white vinegar, pomegranate peel, black coffee (BC), aloe vera peel, and aspirin, had been used as reducing/stabilizing agents to tune the properties of the Fe_3_O_4_ NPs. After utilizing spinach as a precursor and BC as a reducing agent, the X-ray diffraction (XRD) pattern showed cubic magnetite (Fe_3_O_4_) phase. Spherical-shaped nanoparticles (∼20 nm) with superparamagnetic nature indicated by scanning electron microscopy (SEM) monographs, whereas energy-dispersive X-ray gives good elemental composition in Fe_3_O_4_ NPs. A characteristic band of Fe-O at ∼ 561 cm^–1^ was exhibited by the Fourier transform infrared (FTIR) spectrum. X-ray photoelectron spectroscopy (XPS) results confirmed the binding energies of Fe 2p_3/2_ (∼710.9 eV) and Fe 2p_1/2_ (∼724.5 eV) while, Raman bands at ∼310 cm^–1^ (T_2__*g*_), ∼550 cm^–1^ (T_2__*g*_), and 670 cm^–1^ (A_1__*g*_) indicated the formation of Fe_3_O_4_ NPs synthesized using BC extract. The *in vitro* activity of BC-Fe_3_O_4_ NPs significantly inhibited the mycelial growth of *F. oxysporum* both at the third and seventh day after incubation, in a dose-dependent manner. *In vivo* studies also exhibited a substantial reduction in disease severity and incidence by improving plant growth parameters after treatment with different concentrations of BC-Fe_3_O_4_ NPs. The increasing tendency in enzymatic activities had been measured after treatment with different concentrations of NPs both in roots and shoot of tomato plants as compared to the control. Correspondingly, the upregulation of PR-proteins and defense genes are in line with the results of the enzymatic activities. The outcome of the present findings suggests that Fe_3_O_4_ NPs has the potential to control wilt infection by enhancing plant growth. Hence, Fe_3_O_4_ NPs, being non-phytotoxic, have impending scope in the agriculture sector to attain higher yield by managing plant diseases.

## Graphical Abstract

Green synthesis of Magnetite Nanoparticles from Spinach powder and its *in-vitro* and *in-vivo* applications for the management of Tomato wilt.

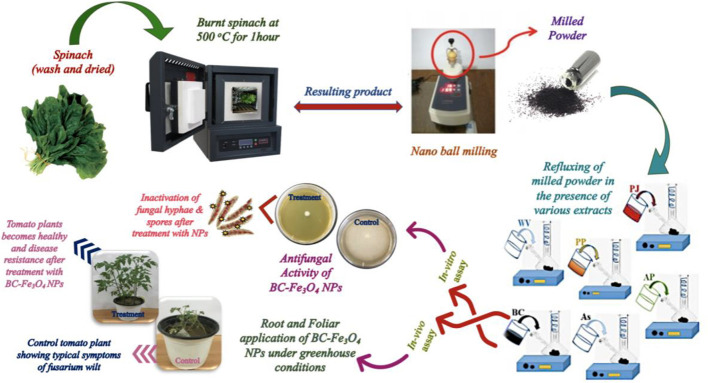

## Highlights

- Magnetite nanoparticles (Fe_3_O_4_ NPs) were green-synthesized using spinach as a starting material.
- XRD results revealed cubic Fe_3_O_4_ NPs using black coffee (BC) extract.
- High dielectric constant (∼575) with low tangent loss (∼ 0.11) at log *f* = 5 was observed for green-synthesized Fe_3_O_4_ NPs using BC extract.
- FTIR results showed the iron oxide characteristic band of Fe-O at ∼561 cm^–1^, and UV-visible analysis revealed Fe_3_O_4_ absorbance band at 282 nm.
- VSM results indicated the superparamagnetic behavior.
- XPS results confirmed the binding energies of Fe 2p_3/2_ and Fe 2p_1/2_ of green-synthesized Fe_3_O_4_ NPs.
- Raman bands at ∼310 cm^–1^ (T_2__*g*_), ∼550 cm^–1^ (T_2__*g*_), and 670 cm^–1^ (A_1__*g*_) showed the formation of Fe_3_O_4_ NPs synthesized using the BC extract.
- SEM results revealed spherical nanoparticles with a diameter of ∼ 20 nm for Fe_3_O_4_ NPs.
- BC-Fe_3_O_4_ NPs significantly inhibited the growth of *Fusarium oxysporum*, both under *in vitro* and *in vivo* assays in a dose-dependent manner.
- Higher levels of defensive biochemicals were observed under various concentrations of Fe_3_O_4_ NPs, in both the roots and shoots of tomato plants.

## Introduction

Magnetic nanoparticles (NPs) are one of the promising nanomaterials with several high-tech applications in material science, engineering, biomedicine, and agriculture (Khandel et al., 2018; Ju et al., 2019; Vu et al., 2020). The pesticide effect of magnetic NPs can be utilized in preventing the growth of several pathogens, i.e., fungi, bacteria, and viruses, etc. (Wu et al., 2016). These NPs are also used as a supplement in animal feed, micronutrients for crops, herbicides, water purifiers, and sewage treatment.

Iron oxide nanoparticles (IONPs) have many advantages in the agricultural field, acting both as antimicrobial agents and plant growth inducers (Siddiqi et al., 2016; Vilardi, 2020). Iron is a key microelement of many physiological processes in plants such as photosynthesis and chlorophyll (Bora et al., 2013; Briat et al., 2015; Vilardi et al., 2019; Vilardi and Verdone, 2020). Iannone et al. (2021) reported that citric acid-coated Fe_3_O_4_ NPs indicated no toxicity in alfalfa and soyabean plants; instead, they act like phytostimulators by promoting plant growth. No side effects were noticed on the growth and development of tomato plants after the treatment of seeds with Fe_3_O_4_ NPs (Lau et al., 2020). Additionally, in contrast, to control, the application of Fe_3_O_4_ NPs enhanced the growth, biomass, moisture content, and availability of phosphorus (P) in *Lactuca sativa* at all concentrations (Zahra et al., 2015). Biogenic Fe_3_O_4_ NPs synthesized exhibited excellent antifungal activity against *Aspergillus flavus* and *F. oxysporum* as compared to the positive and negative control (Salem et al., 2019). Moreover, significant inhibition in mycelial growth and spore germination was detected against different rot-causing fungal strains at the exposure of FeO NPs (Koka et al., 2011). Azhdari et al. (2020) stated that eco-friendly IONPs have potential to replace the hazardous fungicides to enhance food security, as they observed inhibitory effects on the radial growth of *Aspergillus niger* and *Fusarium solani*, respectively. Additionally, the *in vitro* and *in vivo* antifungal activities of chitosan–iron oxide NP studies against *Rhizopus oryzae*-caused fruit rot disease of strawberry substantially reduced the fungus growth and spore formation in a concentration-dependent manner (Saqib et al., 2019).

Tomato (*Solanum lycopersicum* L.) is one of the most important vegetable crops cultivated globally, highly important in terms of consumption and nutritive value, and also utilized as a model plant for research (Rahmatizadeh et al., 2019). Tomatoes are susceptible to more than 200 plant diseases, the utmost of which are caused by fungal pathogens. Both under greenhouse and field conditions, tomato yield is severely impeded by wilt disease caused by *Fusarium oxysporum* f. sp. *lycopersici* (Sacc) Synder et Hansen (Chakraborty et al., 2017). *F. oxysporum* is one of the most challenging soil-borne pathogens and survives for more than decades in soil; 10–50% losses of tomato crop are due to Fusarium wilt. Currently, various strategies are available for the management of Fusarium wilt such as resistant varieties and conventional fungicides, but both methods have some constraints. To overcome these challenges, nanotechnology has suggested alternatives to develop strategies to manage phyto-diseases (Lopez-Lima et al., 2021). As we know, nanotechnology is gaining interest by stipulating solutions for agricultural practices and has the potential to transfigure the prevailing systems of disease management. Nano-pesticides have efficient stability and constancy that enable specific-target delivery and improved formulation by slow degradation of active molecules and improves the selective toxicity by overcoming resistance (Alphandéry, 2020).

Green synthesis of NPs, by utilizing various microbes and green plants, have appeared as feasible nano-factories that are cost-effective and environment friendly (Siddiqi et al., 2016). Green-synthesized NPs offer effective applications for disease management and stress tolerance (Mishra et al., 2017). NPs’ mediated transformation performs an efficient role in the improvement of the plant system through genetic modifications. Explicitly, the utilization of NPs in the area of plant pathology marks certain agriculture-related issues, especially host–pathogen associations. NPs may deliver novel innocuous ways of scientific approaches for the growth and protection of crops (Vega-Vásquez et al., 2020). Green nanotechnological procedures are not only deprived of toxic materials; their stability and rate of production is also fast as compared to microorganism-based synthesis (Shafey, 2020). The synthesis of NPs through the green route by using various plant parts, i.e., leaves, seeds, root, stem, peels, petals, fruits, and flowers, is a reproducible approach as these biomolecules function as stabilizers, reducers, capping agents, and redox-mediators in the synthesis process (Priya et al., 2021). It was also assumed that plant-based NPs are more stabilized in comparison to those synthesized conventionally (Bibi et al., 2019). Some earlier studies reported the synthesis of Fe_3_O_4_ NPs from various plant extracts such as the seed extract of *Phoenix dactylifera*, *Syzygium cumini*, and pomegranate; the leaf extract of *Zanthoxylum armatum*, clover; the fruit extract of *Couroupita. Guianensis;* and the fruit peel extract of *Graptophyllum pictum* and *Garcinia mangostana* (Sari and Yulizar, 2017; Ramesh et al., 2018; Sathishkumar et al., 2018; Bibi et al., 2019; Shafaei et al., 2020; Majid et al., 2021; Srivastava et al., 2021; Yusefi et al., 2021). Furthermore, glucose, sucrose, and other types of reducing sugars have also been used for green synthesis of Fe_3_O_4_ (Sari et al., 2017; Jin et al., 2018; Jain et al., 2019; Dong et al., 2020; Fulaz et al., 2021).

In the current investigation, magnetite NPs (BC-Fe_3_O_4_ NPs) were synthesized by using spinach as a starting material, stabilized by various extracts. We explore the potential of magnetite (Fe_3_O_4_) NPs to subdue the wilt infection in tomato plants. Biocompatible BC-Fe_3_O_4_ NPs have efficiently restricted fungal growth by improving plant growth parameters both under lab and greenhouse conditions. We also measured the phenolic content and activities of defense enzymes [phenyl ammonia-lyase (PAL), peroxidase (PO), and polyphenol-oxidase (PPO)] in the roots and shoots of treated plants. Moreover, the expression of pathogenesis-related protein and defense genes were also studied to evaluate the efficacy of BC-Fe_3_O_4_ NPs in the infected plant.

## Materials and Methods

### Collection of Materials and Procurement of Fungal Culture

Fresh spinach (*Spinacia oleracea*) was bought from a local vegetable market in Lahore, Pakistan, and was washed thoroughly with deionized (DI) water to remove the dust materials. Six different extracts (natural, medicine based), used for the synthesis of NPs are tabulated in Table 1. To prepare the extracts, DI water was used as a solvent. *Fusarium oxysporum* f. sp. *lycopersici* (FOL), pure slant with accession no: IAGS-1322, was acquired from FCBP (First Culture Bank of Pakistan, Department of Plant Pathology, University of the Punjab, Lahore, Pakistan). The fungal strain was retained, sub-cultured periodically, and conserved on potato–dextrose–agar (PDA) medium in culture tubes at 4°C for later use.

**TABLE 1:** Extracts used in the preparation of green-synthesized iron oxide nanoparticles (IONPs).

Sr#	Extracts	Abbreviation	pH
1	Pomegranate juice	PJ	2
2	White vinegar	WV	3
3	Pomegranate peel	PP	4
4	Black coffee	BC	5
5	Alovera peel	AP	5.5
6	Aspirin	As	7

### Green Synthesis of Iron Oxide Nanoparticles

**FIGURE 1:**
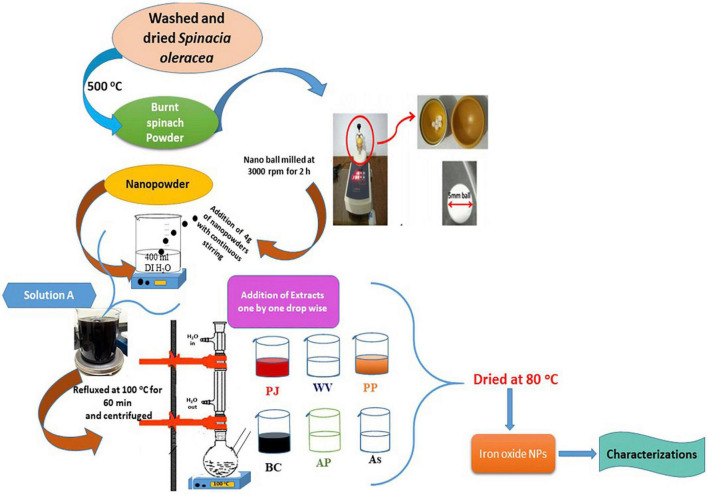
Schematic showing the green synthesis of iron oxide nanoparticles (IONPs) using spinach and various extracts.

Spinach (*S. oleracea*) was used as a starting material for the synthesis of Fe_3_O_4_ NPs. Dried spinach leaves (5 kg) were kept in a furnace at 500°C for 1 and 2 h, separately, to get powder. Burned spinach powders were nano ball-milled at 3,000 rpm for 2 h to obtain fine nanopowder. Four grams of nanopowder (burnt and nano ball-milled spinach) was dissolved into 400 ml of DI water under vigorous stirring. This starting amount of powder was selected to make a homogenous and saturated solution to be processed further. This solution acted as a stock solution and was named “A.” Six different solutions were prepared using six extracts (Table 1). The molarity of each solution was maintained as 0.01 M with 10 ml of extract solution. All six solutions were taken in separate glass flasks for refluxing. Solutions were refluxed at 100°C for 60 min, bubbled with nitrogen gas. The resulting product was separated and centrifuged. The green-synthesized Fe_3_O_4_ NPs were dried at 80°C for further characterization. The schematic is shown in Figure 1.

### Characterization of Green-Synthesized Iron Oxide Nanoparticles

Structural investigations of IONPs were performed by using an X-ray diffractometer (Bruker D-8 Advance). Shimadzu FTIR spectroscopy (IR Tracer –100) was used to analyze various chemical bonds. UV-visible spectrum was obtained using a Shimadzu UV-visible spectrophotometer (UV-1800). VSM (Lake Shore’s 7407) was used for the analysis of the magnetic response of NPs, while SEM and energy-dispersive X-ray (EDX) were used to investigate the morphology and content of various elements. XPS analyses were obtained by the Thermo Fisher Scientific ESCALAB system. Raman spectroscopy was used to investigate various phases of magnetic NPs.

### Ferric-Reducing Antioxidant Power and Total-Polyphenol Analysis of Extracts

The reducing power of the spinach (precursor) and all extracts involved in the synthesis process, was estimated by the ferric-reducing antioxidant power (FRAP). This test estimates the potential of antioxidants to reduce ferric iron to ferrous iron. The extract solution (1 ml) was added to equal amounts (2.5 ml) of sodium phosphate buffer (pH 6.6 and 0.2 M) and potassium ferricyanide solution (1% w/v). The mixture was vortexed and incubated at 50°C for 20 min with constant shaking. The resultant supernatant was centrifuged at 3,000 rpm after adding 2.5 ml trichloroacetic acid (10% w/v). Afterward, the volume of the solution was increased up to 3 ml by adding 0.5 ml of ferric chloride (0.1% w/v) and 2.5 ml of DI water. The absorbance, of the reaction mixture, was measured at λ 593 nm against blank, while the total phenolics in extracts were calculated by the Folin–Ciocalteu method by following the protocol of Singh et al. (2002). Briefly, 1 ml of extract solution was added to 2.5 ml of Folin–Ciocalteu reagent (10% w/v); after 5 min, 2.0 ml of sodium carbonate (75%) was added to the solution and left for 30 min at room temperature. The absorbance was measured at 765 nm with a UV-visible spectrophotometer.

### Pathogenicity Test for Procured Fungal Strain

To check the formae specials (f. sp.) of the procured strain, a virulence test of the strain was carried out against three susceptible tomato varieties (Fine-Star, Early Boy, and Rio-Grande). Twenty-five-day-old healthy seedlings were inoculated with fungal strain by using the root dip method. Briefly, roots were carefully uprooted, washed, and submerged in conidial suspension for 30 min. For control, seedlings were dipped in sterile distilled water only. Next, five seedlings (control and treated) per pot were transplanted to new pots containing sterilized sandy loam soil (1:1). Pots were kept in a greenhouse with varying temperatures of 25–30°C. Plants were watered daily and fertilized with Nitrogen:Phosphorus:Potassium (NPK) (15:15:15) once during the growth period. Symptoms were visible after 15–25 days, while disease severity was measured up to 45 days. The discoloration of the stem vascular tissues was examined by slashing the stem of the infected plant (Nirmaladevi et al., 2016).

### Antifungal Activity of Black Coffee–Magnetite Nanoparticles

To explore the antifungal activity of BC-Fe_3_O_4_ NPs, the mycelial inhibition method was followed. *In vitro* antifungal activity was checked by preparing PDA plates with various concentrations of BC-Fe_3_O_4_ NPs (0.01, 0.5, 1.5, 2.5, 5, 7.5, 10, 12.5, and 15 μg/ml), while control and the fungicide plate received a distilled water and fungicide solution. After incubation for 24 h, plugs of uniform size (4 mm) from 7 days of the culture of FOL were shifted to the center of each plate and incubated at 28°C under dark conditions. All experiments were performed in triplicate. At the end of the incubation period, i.e., 3 and 7 days, fungal mycelial growth was measured by using Eq. 1 (Kim et al., 2012):


(1) $$InhibitionRate\left(\%\right)=\left(\frac{R-r}{R}\right)\times 100$$

Where,

*R* = radial growth of fungal mycelia in control plates

*r* = radial growth of fungal mycelia in treated plate

### Greenhouse Experiment to Assess the Efficacy of Black Coffee–Magnetite Nanoparticles

An *in vivo* trial was conducted to check the efficacy of BC-Fe_3_O_4_ NPs under greenhouse conditions. For this, sterilized plastic pots of 60 in. length and 15 cm diameter were filled with steam-sterilized sandy clay soil at 0.5 kg soil per pot. The fungal inoculum was prepared by adjusting the final concentration of the spore’s suspension to 1 × 10^6^ spore/ml in sterilized water. Each pot was inoculated, with 50 ml of spore suspension, and kept at 30°C in a greenhouse for 1 week before sowing; soil moisture was maintained. The roots of young tomato seedlings (cv. Rio-Grande: 25–30 days old) were dipped in various concentrations of BC-Fe_3_O_4_ NPs (0.01, 0.5, 1.5, 2.5, 5, 7.5, 10, 12.5, and 15 μg/ml) for 2 h (Ashraf et al., 2020), while for control and fungicide (Nativo), roots were treated with distilled water and fungicide (Nativo) solution, respectively. Afterward, seedlings were shifted to the pots treated with fungal inoculum at the rate of five plants per pot. After 2 weeks of transplanting, tomato plants were sprayed with two foliar sprays, with an interval of 10 days between each spray. Different physiological parameters were recorded after 45 days of growth, including disease severity and incidence and growth parameters (plant height, biomass, root, and shoot length). The disease severity was measured according to the scale used by Nirmaladevi et al. (2016) for Fusarium wilt. The disease scale was ranged from 0 to 100% (0: healthy plants; 25%: initial symptoms showing leaf chlorosis; 50%: the initiation of wilting with severe chlorosis; 75%: severe wilting with leaf chlorosis; 100%: leave necrosis with a completely wilted plant. Fresh biomass (g) was estimated by removing excess moisture by filter paper, while dry biomass (g) was determined by drying the same seedlings in a hot oven for 72 h at 40°C. Percentage disease incidence was calculated by using Eq. 2 (Vincent, 1947).


(2) $$\text{Dise}\mathrm{ase}\mathrm{Incidence}\left(\%\right)=\left(\frac{\mathrm{Number}\,\mathrm{of}\,\mathrm{infected}\,\mathrm{plants}}{\mathrm{Total}\,\mathrm{number}\,\mathrm{of}\,\mathrm{plants}}\right)\times 100$$

### Estimating Total Phenolic Content and Stress Enzymes

The total phenolic content and stress enzymes were quantified on the second day after the second foliar spray of BC-Fe_3_O_4_ NPs. The roots and shoots of tomato plants from each treatment were detached for measuring PAL, POD, and PPO by using the following protocols.

### Measurement of Total Phenolic Content

Total phenolic content was measured by following the protocol of Zieslin and Zaken (1993). For this, initially, a clean test tube was filled with 5 ml of distilled water. Afterward, 250 μl of 50% Folin–Ciocalteu reagent, along with 1 ml of methanolic extract, was added to the test tube and left for half an hour under dark conditions. Furthermore, the reaction mixture was incubated for 10 min by adding 1 ml of 50% sodium carbonate solution. The absorbance value, at 725 nm, was measured using catechol as standard. The values of the total phenolic content were calculated by comparing it with a standard curve in terms of “μg catechol mg^–1^.”

### Measurement of Stress Enzymes

To quantify enzymes, a supernatant was prepared by crushing plant material (∼1 g) with ice-cold phosphate buffer (100 mM: pH = 7) in a pre-chilled mortar. Subsequently, the homogenized plant material was centrifuged at 5,000 rpm for 15 min. Pure supernatant was collected and further used for enzyme estimation.

### Measurement of Peroxidase

POD enzyme activity was estimated by using guaiacol as substrate according to the method proposed by Fu and Huang (2001). Briefly, hydrogen POD (100 μl) was melded with guaiacol reagent (250 μl); thereafter, 10 ml of sodium phosphate buffer (10 mM with pH = 6) was added to this solution. Finally, enzyme extract (3 ml) was added and incubated for 5 min at room temperature; afterward, absorbance was measured at 470 nm. Enzyme activity was denoted as Δ470 nm g^–1^ fresh weight minute^–1^.

### Measurement of Polyphenol-Oxidases

The activity of the PPO enzyme was measured by using catechol as a substrate. The reaction mixture was prepared by mixing 150 μl catechol solution (0.1 M) with 1.5 ml of phosphate buffer (10 mM with pH = 6). Next, 200 μl of enzyme solution was added to the above reaction mixture and left for 1 h at room temperature. Later, absorbance was measured at 495 nm. PPO enzyme activity was denoted as Δ495 nm mg^–1^min^–1^ protein (Mayer et al., 1966).

### Measurement of Phenylalanine Ammonia Lyase

For the quantification of phenylalanine ammonia lyase (PAL), reaction mixture containing 0.03 M L-phenylalanine (250 μl) and enzyme extract (200 μl) in total volume of 2.5 ml of sodium borate buffer (pH = 8). The reaction mixture was left for 1 h at 37°C in a water bath. Afterward, 1.0 M trichloroacetic acid (0.5 ml) was added to this solution and absorbance was measured at 290 nm. PAL enzyme activity was indicated as micrograms of trans-cinnamic acid mg^–1^h^–1^ protein (Burrell and Rees, 1974).

### Expression of Genes in Tomato Plants Quantified by qRT-PCR

Changes in the expression of pathogenicity- and defense-related proteins were evaluated by qRT-PCR in tomato roots exposed to BC-Fe_3_O_4_ NPs. The fresh root tissues of tomato plants, treated with higher concentrations, i.e., 10, 12.5, and 15 μg/ml of BC-Fe_3_O_4_ NPs, were harvested after 24, 48, and 72 h of exposure to a second foliar spray of NPs and kept at −80°C. Procedures regarding total RNA isolation, synthesis of complementary DNA (cDNA) and gene expression using qRT-PCR have been followed by using a protocol of Ma et al. (2013) with minor modifications. Briefly, harvested plant roots were crushed in liquid nitrogen for the isolation of total RNA by using the Ribosin™ plant kit (Gene All, Seoul, South Korea) according to manufacturer’s instructions. The RNA concentrations were quantified by nanodrop spectrophotometry (NanoDrop 1000; Thermo Fisher Scientific, Waltham, MA, United States). The cDNA was synthesized from the total RNA using GScript First-Strand Synthesis Kit (GeneDireX, New Taipei, Taiwan) according to the manufacturer’s protocol. Primer specificities were confirmed by agarose gel electrophoresis of the RT-PCR products. Eventually, 100 ng/μl cDNA was used as a template to run qRT-PCR in PikoReal^®^ Real-Time 96 PCR System (Thermo Fisher Scientific, Waltham, MA, United States) with SYBR Green qPCR Master Mix (2X) (Thermo Fisher Scientific, Waltham, MA, United States). The amplification program was adjusted according to the following thermocycling conditions for all reactions starting with initial denaturing step of 95°C for 10 min (optics-off), followed by a loop of 40 cycles at 95°C for 15 s (optics-off), and 60°C for 30 s (optics-on) and temperature ramp from 60 to 95°C at 0.2 C/s for melting-curve analysis. Actin was used as an internal standard and housekeeping gene. All qRT-PCR reactions were amplified in triplicates. The threshold-cycle (Ct) value was acquired by using Thermo Fisher Scientific PickoReal™ software 2.1 (Thermo Fisher Scientific, Waltham, MA, United States). A total volume of 20 μl was used for qRT-PCR. Quantification results were analyzed by the 2^–ΔΔCt^ method to calculate the relative level of gene expression with/without BC-Fe_3_O_4_ NP treatment (Livak and Schmittgen, 2001). A list of primer sequences used in this study is provided in Table 2.

**TABLE 2:** A list of primers sequence for qRT-PCR analysis.

Primers	Primer Sequence (5′^–^3′)
ACTIN-F	CATTGTGCTCAGTGGTGGTTC
ACTIN-R	TCTGCTGGAAGGTGCTAAGTG
PR2-F	GTTTACTGCGCTACCTGGGA
PR2-R	CCTGTGTTGGTCACCCTCAA
PAL-F	TTATTAGGTTCTTGAATGCTGGAGT
PAL-R	CAAACACGGGGTGATGTTGC
PPO-F	CTTCTGTGACTAAGCTCCGTATT
PPO-R	AGGGTTATCAGGTTGTGTCTTATC
POD-F	ACGGAGCAAGCGACAATTGACAAC
POD-R	CGATTGATTCACCGCAAAGCTCGT

*F, forward primer; R, reverse primer.*

### Statistical Analysis

Statistical analysis has been performed by using computer-aided software Statistic 8.1. All experiments were independently repeated in triplicates, and the results were interpreted as mean ± standard deviation (SD). The differences among the groups were retrieved by using the analysis of variance test (one-way ANOVA). Different superscripted letters indicate substantial differences among treatments determined by LSD Fisher’s test (*p* < 0.05).

## Results and Discussion

### Quantification of Ferric-Reducing Antioxidant Power and Total Polyphenols in Extracts

The presence of FRAP and total polyphenols in spinach and other extracts used for the synthesis of Fe_3_O_4_ NPs is shown in Supplementary Figures 1A,B. The presence of Fe^2+^ in cells may lead to toxic effects being involved in the Fenton reaction, generating OH after reaction with hydrogen peroxide that will initiate the oxidation process (Halliwell, 2008; Richards et al., 2015). FRAP analysis gives information about the ability of the sample to reduce a ferric ion to ferrous ion, so it determines the antioxidant activity of biological samples (Pulido et al., 2000; Guo et al., 2003). All extracts showed the reducing power of Fe^+2^, while the highest activity was observed for black coffee (BC), followed by pomegranate juice (PJ), aloe vera peel (AP), pomegranate peel (PP), white vinegar (WV), and aspirin (As) compared to standard spinach (Supplementary Figure 1A). It can be presumed that certain secondary metabolites such as anthocyanins, caffeic acid, and hydrolyzable tannins available in BC extract might be responsible for this high activity. Similarly, Aryal et al. (2019) performed the FRAP assay for different plant extracts, stating that the reducing power of Fe^+2^ depends on the concentration of extracts. The correlation between antioxidant activity and total phenolic contents coincides with the findings of Tzulker et al. (2007). Likewise, in FRAP analysis, a higher amount of polyphenols was also observed in BC extract (Supplementary Figure 1B). Polyphenolic compounds are commonly available in plants, and reveal antioxidant activity, which is primarily due to redox properties (Koshelev et al., 2020). Generally, various mechanisms are involved by phenolic compounds to exert antioxidant activity such as the direct elimination of reactive oxygen species (ROS), by restricting the enzymatic activity and chelation of metal ions like iron by inhibiting the series of oxidative reactions (Loizzo et al., 2019).

### Structural/Phase Analysis of Green-Synthesized Iron Oxide Nanoparticles

**FIGURE 2:**
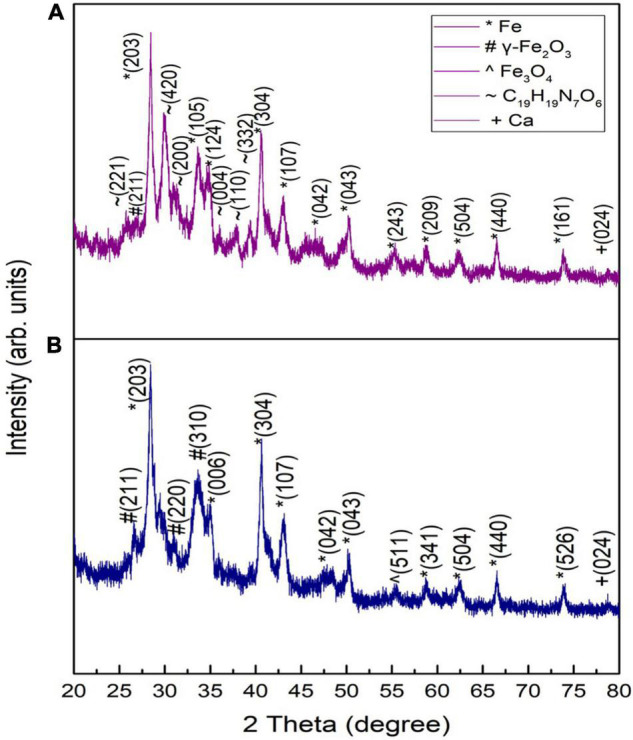
XRD patterns of raw spinach burnt at 500°C for **(A)** 1 h and **(B)** 2 h.

The structure, phase, crystallite size, and crystallinity of the IONPs were determined by XRD patterns ranging from 20 to 80 at a 2θ angle. Figure 2A shows the XRD pattern of spinach (dried/burnt) at 500°C for 1 h.

Peaks appearing at 28.40, 33.62, 39.32, 40.55, 43.02, 50.23, 55.26, 58.77, 62.38, 66.55, and 73.7° matched with the standard XRD spectrum of Fe NPs as per JCPDS card No: 96-411-3931. A peak appearing at an angle of 26.55° matched well with γ-Fe_2_O_3_ according to JCPDS card no. 25-1402, while peaks appearing at 25.75, 30.01, 31.16, and 37.8° corresponded to C_19_H_19_N_7_O_6_ (i.e., folic acid) according to JCPDS no. 29-1716. The peaks of C_19_H_19_N_7_O_6_ did not appear by increasing the combustion time to 2 h (Figure 2B). Such a response showed the complete decomposition of folic acid. As folic acid does not have any apparent melting temperature, the decomposition can take place in three stages. In the first stage, an anhydrous sample was formed due to the loss of adsorbed water. In the second stage, the glutamic acid moiety vanished, degrading other elements of folic acid. The basic component amide was left when glutamic acid broke down from folic acid. After that, p-aminobenzoic acid and pterin decomposed. Black residue, i.e., traces of iron and its compounds, was found in the furnace due to the burning of spinach (Vora et al., 2004).

**FIGURE 3:**
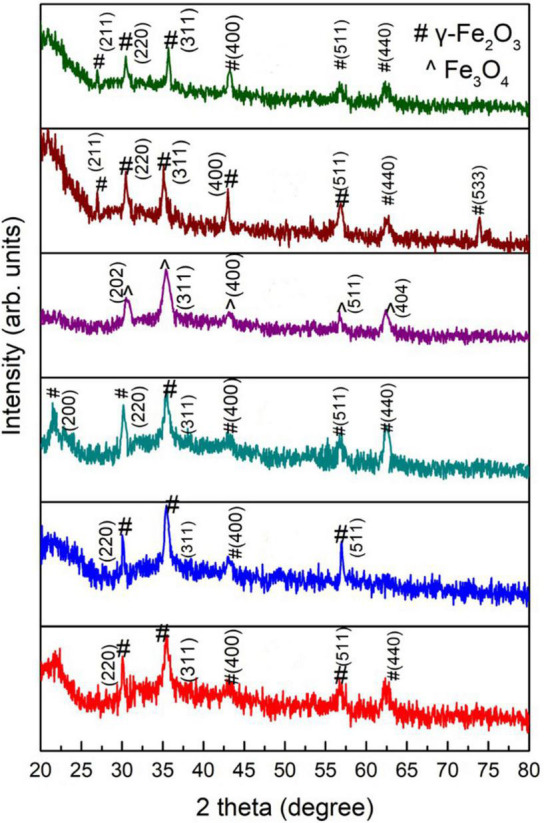
XRD patterns of green-synthesized IONPs synthesized by using spinach and extracts: **(A)** PJ, **(B)** WV, **(C)** PP, **(D)** BC, **(E)** AP, and **(F)** As extracts.

The XRD patterns of green-synthesized IONPs by using various extracts are shown in Figure 3. The XRD pattern of Fe_3_O_4_ NPs synthesized using PJ showed peaks corresponding to (220), (311), (400), and (511) planes of γ-Fe_2_O_3_ (Figure 3A). These peaks matched with the standard XRD spectrum of γ-Fe_2_O_3_ NPs (JCPDS card No: 00-004-0755). PJ is very rich in polyphenols (Irshad et al., 2017), which act as reducing agents for Fe ions, generating IONPs. Further, polyphenols also stabilize the synthesized NPs by avoiding agglomeration as well as oxidation (Mystrioti et al., 2016).

An XRD analysis of NPs synthesized using WV shows the formation of phase pure γ-Fe_2_O_3_ (Figure 3B). Crystallographic planes (220), (311), (400) and (511) matched well with the JCPDS card no. (00-004-0755). WV extract is an aqueous solution of acetic acid and other trace elements. Acetic acid generates linkage among iron-rich canters to form iron-acetic species, i.e., an anchoring of acetic anions onto iron is produced. These anions bind with iron centers during chelating, helping in the production of iron oxide (Hong et al., 2008). In contrast to PJ, the peel of pomegranate contains more polyphenols. Pomegranate peel (PP) consists of different phenolic compounds, i.e., the derivatives of ellagic acid and ellagic such as punicalagin. Besides reducing nature, these complexes play a major role in the stability of synthesized NPs (Irshad et al., 2017). (200), (220), (311), (400), (511), and (440) planes of maghemite (γ-Fe_2_O_3_) were observed for NPs synthesized using PP extract (Figure 3C). Magnetite (Fe_3_O_4_) NPs were obtained using BC during the synthesis process (Figure 3D). Peaks corresponded to (202), (311), (400), (511), and (404) planes of cubic magnetite (Fe_3_O_4_) (JCPDS card no. 96-900-2317). The major constituents of BC are caffeine and tannin. Tannins are composed of polyphenolic compounds (non-toxic) that are reducing and stabilizing agents for the synthesis of BC-Fe_3_O_4_ NPs. The phenolic-OH, as well as ortho-dihydroxy phenyl groups present in the chemical structure of tannins, is responsible for the complex formation with iron. These groups also participate in redox reactions (Herrera-Becerra et al., 2010). Therefore, it can be predicted that the synthesis of Fe_3_O_4_ nanoparticles is governed by the tannins present in BC extract. Synthesis was also performed using AP extract. AP acts as a complex agent in green synthesis and thus can be used to prepare nanocrystalline metal-oxides (Phumying et al., 2013). Nanoparticles synthesized using AP extract exhibited γ-Fe_2_O_3_ NPs as shown in Figure 3E. Phenolic compounds, present in AP extract, are good capping agents and are helpful in the stabilization of NPs by avoiding aggregation (Saif et al., 2016). NPs prepared using aspirin (As) extract showed the formation of phase-pure γ-Fe_2_O_3_ NPs (Figure 3F). Aspirin, known as acetylsalicylic acid, may act as a capping as well as a reducing agent in the synthesis of NPs (Bahram and Mohammadzadeh, 2014).

Crystallite size (*t*) and dislocation density (δ) were calculated using Eqs 3 and 4, respectively (Matacotta and Ottaviani, 1995) and are plotted in Supplementary Figure 2.


(3) $$t=\frac{k\,\lambda }{\beta \,\cos\theta }$$

(4) $$\delta =\frac{1}{t^{2}}$$

where *k* is the shape factor taken as 0.9, λ is the wavelength of Cu kα source, β is full width at half-maximum, and θ is the diffraction angle of highest intensity peak.

A variation in crystallite size was observed with the variation in various extracts used during synthesis. BC played a critical role not only in achieving the Fe_3_O_4_ phase of iron oxide (Figure 3D) but also in achieving a small value of crystallite size.

### Dielectric Analysis of Green-Synthesized Magnetite Nanoparticles

The frequency-dependent dielectric constant of magnetite nanoparticles (Fe_3_O_4_ NPs) was obtained by an impedance analyzer using Eq. 5, whereas the loss factor was calculated using Eq. 6 (Barsoukov and Macdonald, 2005).


(5) $$\varepsilon =\frac{C\times d}{\varepsilon_{o}\times A}$$

(6) $$\tan\delta =\frac{1}{2\,\pi \,f\,\varepsilon \,\varepsilon_{o}\,\rho }$$

where *C* indicates capacitance, *d* is used for thickness, *A* for the area, **upvarepsilon*_*o*_* is the permittivity of free space, and ρ is resistivity.

**FIGURE 4:**
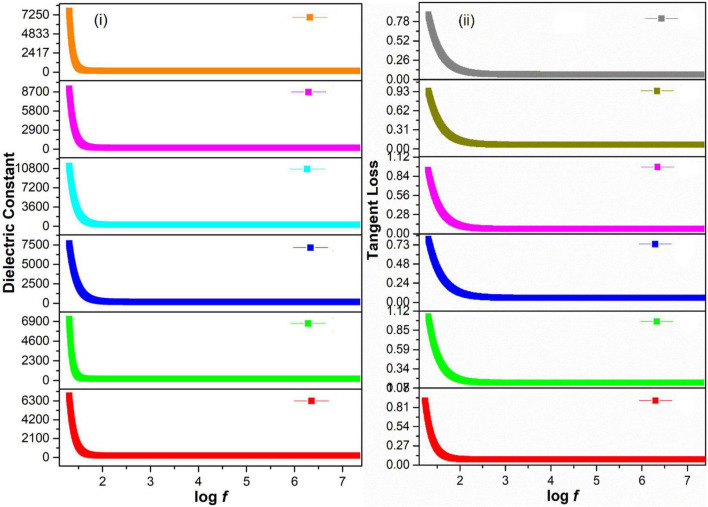
**(i)** Dielectric constant and **(ii)** Tangent loss of green-synthesized IONPs by (A) PJ, (B) WV, (C) PP, (D) BC, (E) AP, and (F) As extracts.

Figures 4i,ii showed a decrease in dielectric constant and tangent loss with increasing frequency at all synthesis conditions. Possible polarizations such as ionic, dipoles, and electronic may occur under the effect of an applied electric field. At low frequencies, space charge polarization dominates, whereas electronic and ionic polarizations are dominant at high frequencies. A high dielectric constant, in low frequencies, was observed due to space charge polarization (SCP). SCP comes from accumulated charges present at grain boundaries. On the other hand, dipoles do not respond in the high-frequency region, giving a low value of the dielectric constant. This type of behavior can be explained by Koop’s theory and the Maxwell–Wagner model that indicates grain boundaries and grains are active in low frequencies and high frequencies, respectively (Koops, 1951; Riaz et al., 2013; Shah et al., 2014; Tahir et al., 2019). Tangent loss is the lag of polarization as a result of the applied alternating field. It could be described by induced defects present in a material. The Maxwell–Wagner model tells about tangent loss (relatively high tangent loss in low-frequency region is caused by defects in material), whereas, in the high-frequency region, the polarization ability of these imperfections lags externally applied field, decreasing the tangent loss (Bashir et al., 2018).

**FIGURE 5:**
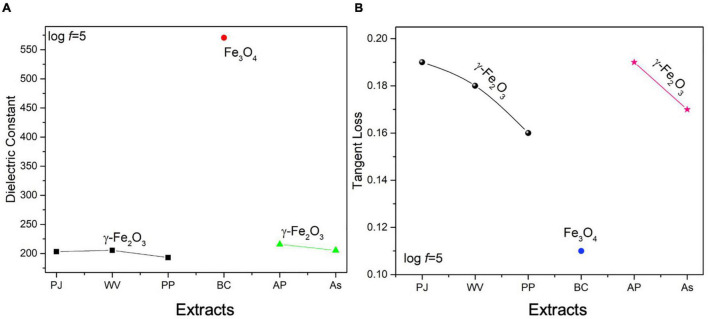
Variation in **(A)** dielectric constant, **(B)** tangent loss of IONPs at log *f* = 5 FTIR, and UV-visible analysis of green-synthesized IONPs.

The variation in dielectric constant and tangent loss under various synthesis conditions is shown in Figures 5A,B. The high value of dielectric constant (∼575 at log *f* = 5) with low tangent loss (∼0.11) was observed for the sample prepared with extract of BC. Such high dielectric polarization response with BC extract is attributed to the formation of phase-pure Fe_3_O_4_ as shown in the XRD result (Figure 3D). The high value of a dielectric constant is attributed to the presence of both Fe^3+^ and Fe^2+^ cations in the Fe_3_O_4_ phase of iron oxide. A heterogeneity in the Fe_3_O_4_ structure arises because of the existence of Fe^2+^ cations that give high polarization, leading to a higher value of dielectric constant (Ni et al., 2009; Nithya and Kalai Selvan, 2011).

**FIGURE 6:**
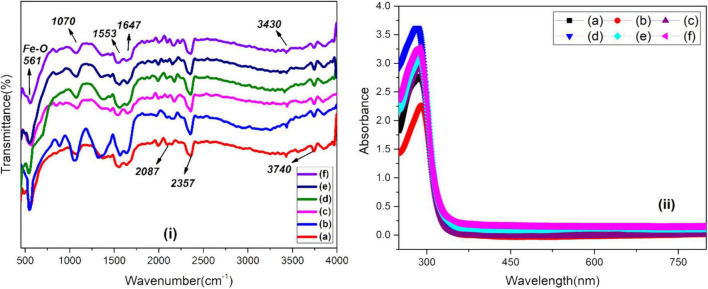
**(i)** FTIR and **(ii)** UV-visible spectra of green-synthesized IONPs by using (A) PJ, (B) WV, (C) PP, (D) BC, (E) AP, and (F) As extracts.

The FTIR spectra of green-synthesized IONPs by using different extracts is shown in Figure 6i. The presence of phytochemicals in extracts can play a vital role as stabilizing and reducing agents. The characteristic band of Fe-O appeared at ∼ 561 cm^–1^ agreed well with earlier literature (Raees et al., 2019). The band at 1,070 cm^–1^ showed the presence of C-N stretching, whereas bands appearing at 1,553 and 1,647 cm^–1^ can be ascribed to C = C stretching vibration (i.e., aromatic rings/phenolic group), N-H of amine vibration, C = O of amides and carboxylic groups. A small band present at 3,433 cm^–1^ could be associated with the OH of the compounds of phenolic. The presence of the phenolic feature indicates the capping effect on the surface of Fe_3_O_4_ NPs. The absorbance band at ∼2,087 cm^–1^ is attributed with alkyne groups of the phytoconstituents of extracts (Carmona et al., 2017). The bending vibration of the H-O-H group is present between 1,672 and 1,367 cm^–1^, whereas feeble bands in the range of 3,028–3,400 cm^–1^ are characteristic of the O-H group stretching vibration (Malega et al., 2018). The absorption band at 2,352 cm^–1^ corresponds to CO_2_. Hence, the presence of phytochemicals, phenolic groups and tannins, etc., were found to be responsible for the stabilization of IONPs through green synthesis. The stability and formation of IONPs green-synthesized using various extracts was also examined using UV-visible analysis as shown in Figure 6ii. Fe_3_O_4_ NPs, synthesized using BC extract, showed maximum characteristic surface plasmon absorbance at 282 nm that matched well with reported values for IONPs (Awwad and Salem, 2013; Asoufi et al., 2018; Rahmani et al., 2020).

### Magnetic Response of Green-Synthesized Iron Oxide Nanoparticles

**FIGURE 7:**
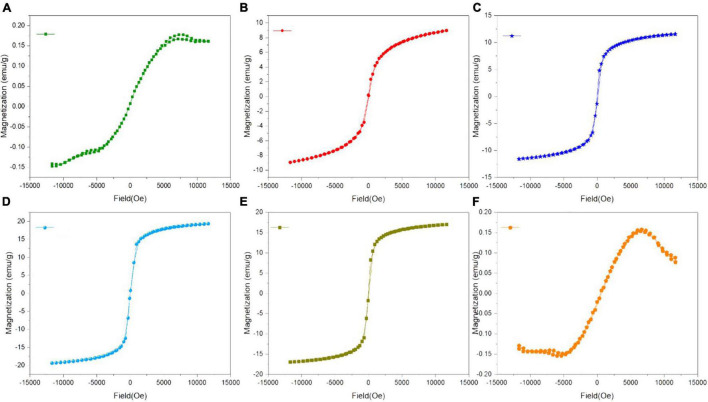
M-H loops of green-synthesized IONPs by using extracts **(A)** PJ, **(B)** WV, **(C)** PP, **(D)** BC, **(E)** AP, and **(F)** As.

The M-H loops of green-synthesized IONPs by using various extracts are shown in Figures 7A–F. The soft ferromagnetic response was observed with all the synthesis conditions. However, the high saturation magnetization (∼19.30 emu/g) and low coercivity (∼14 Oe) observed for IONPs green-synthesized using BC extract indicated the superparamagnetic nature (Figure 7D).

Superparamagnetic behavior arises when the size of a single domain becomes so small that thermal energy can easily overcome the anisotropy energy barrier, while hysteresis loops appear after the application of an external magnetic field to the NPs and saturation magnetization (M_*s*_) declined from a plateau state to zero (El-Boubbou et al., 2019). Based on these characteristics, it can be stated that IONPs have the potential of facilitating agricultural applications including the targeted delivery of nutrients and controlled release of pesticides. Moreover, the decrease in magnetization due to the well-dispersed, ultrafine nature of IONPs, additional surface spin, cation distribution, and surface disarray results in varying the Ms of the magnetic product (Lakshminarayanan et al., 2021). The variation in saturation magnetization (M_*s*_) and coercivity (H_*c*_), under various synthesis conditions, is shown in Supplementary Figures 3A,B.

### X-Ray Photoelectron Spectroscopy Analysis of Green-Synthesized Iron Oxide Nanoparticles

**FIGURE 8:**
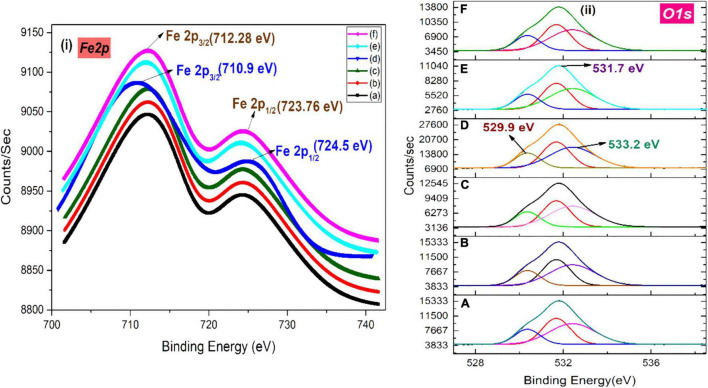
XPS spectra of **(i)** Fe 2p and **(ii)** O 1s of green-synthesized IONPs by using (A) PJ, (B) WV, (C) PP, (D) BC, (E) AP, and (F) As extracts.

The XPS analysis of IONPs synthesized using spinach as precursor along with various extracts is depicted in Figures 8i,ii. Binding energy peaks of Fe 2p_3/2_ and Fe 2p_1/2_ at ∼712.28 and 723.76 eV were observed using various extracts (Figure 8i). Grosvenor et al. (2004) reported similar values for iron compounds, confirmed the maghemite phase of iron oxide. However, the presence of binding energy peaks of Fe 2p_3/2_ and Fe 2p_1/2_ at ∼710.9 and ∼724.5 eV indicated the formation of Fe_3_O_4_ NPs, which was perceived in the case of BC extracts. The peaks of split spin-orbit Fe 2p are wide due to the less chemical shift between Fe^2+^ and Fe^3+^ (Tiwari et al., 2007). Figure 8ii represents the O1s core level; peaks at 529.9 and 531.7 eV can be associated with the existence of O^–2^ and OH^–^ species present at the iron oxide surface, respectively, and another peak at 533.2 eV is the indication of adsorbed H_2_O molecules (Medina-Llamas and Mattia, 2019).

### Raman Spectra of Green-Synthesized Iron Oxide Nanoparticles

**FIGURE 9:**
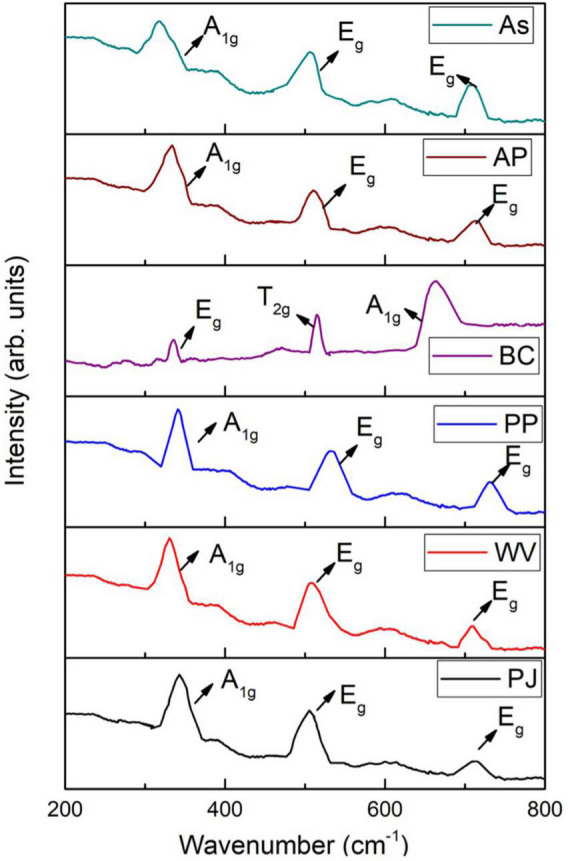
Raman spectra of iron oxide nanoparticles (IONPs) using various extracts.

Raman spectroscopy was used to investigate various phases of IONPs, i.e., hematite (α-Fe_2_O_3_), maghemite (γ-Fe_2_O_3_), and magnetite (Fe_3_O_4_). The Raman spectra of green-synthesized IONPs are shown in Figure 9. IONPs synthesized by using PJ, WV, PP, AP, and As extracts exhibited three Raman active phonon modes at ∼360 cm^–1^(T_2g_), ∼510 cm^–1^(E_*g*_), and ∼700 cm^–1^(A_1g_), respectively. The presence of these modes indicates the formation of maghemite (γ-Fe_2_O_3_) NPs (Legodi and de Waal, 2007; Jubb and Allen, 2010; Slavov et al., 2010) as observed in XRD results presented in Figure 3. However, the presence of Raman bands at ∼310 cm^–1^ (T_2g_), ∼550 cm^–1^ (T_2g_), and 670 cm^–1^ (A_1g_) indicated the formation of Fe_3_O_4_ NPs. So, the NPs synthesized by using BC extract showed the BC-Fe_3_O_4_ NPs. These bands matched well with the previously reported Raman modes for the magnetite phase (de Faria et al., 1997; Jubb and Allen, 2010; Schemme et al., 2015).

### Surface Morphology of Green-Synthesized Iron Oxide Nanoparticles

**FIGURE 10:**
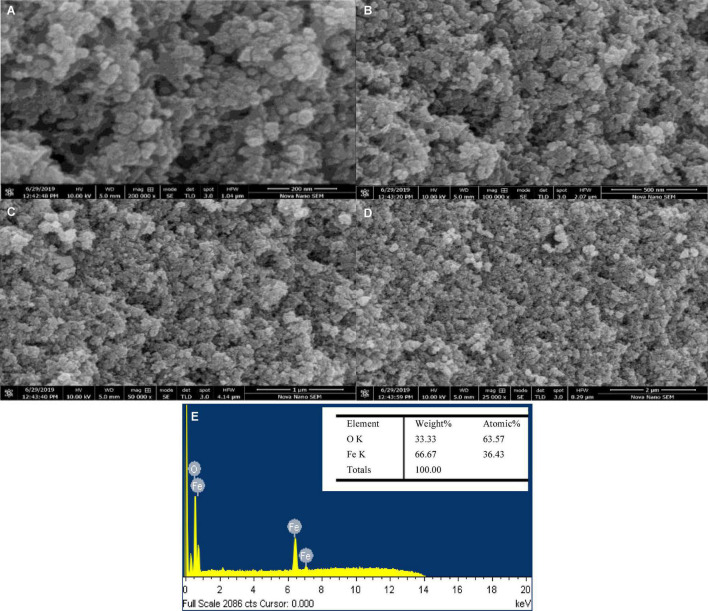
SEM images **(A–D)** of green-synthesized BC-Fe_3_O_4_ NPs at different magnifications (200 and 500 nm and 1 and 2 μm). EDX spectra **(E)** of BC-Fe_3_O_4_ NPs.

The SEM monographs of green-synthesized IONPs with different magnifications by using various extracts is shown in Figures 10A–D and Supplementary Figures 4A–E. The microscopic images (Figures 10A–D) revealed that the spherical-shaped NPs with well-defined grain boundaries and a diameter of ∼20 nm were observed by using BC (Khatami et al., 2017; Beheshtkhoo et al., 2018), whereas the SEM images of IONPs with other extracts such as PJ and WV showed agglomerated structures with no specific morphology. Correspondingly, soft agglomeration was observed using AP and aspirin (As) extracts (Supplementary Figure 4-d and e). Furthermore, the size of IONPs synthesized by using other extracts were found to be ∼30 nm (PJ), ∼50 nm (WV), ∼20 nm (PP), ∼25 nm (AP), and ∼28 nm (aspirin), respectively. The EDX spectra of green-synthesized IONPs by using BC and other extracts are shown in Figure 10E and Supplementary Figures 5A–E. The shape and small size of BC- Fe_3_O_4_ NPs were due to the agglomeration that existed among the particles due to magnetic attractions or van der Waals forces (Yusefi et al., 2020). Furthermore, the occurrence of the hydroxyl group in the leaf extract of coffee could induce agglomeration (Kurang and Kamengon, 2021). The EDX spectrum of Fe_3_O_4_ NPs showed peaks at 0.8, 6.2, and 7.2 keV that were coupled with iron-binding energies (Basavegowda et al., 2014). Based on the well-defined surface morphology and superparamagnetic nature, Fe_3_O_4_ NPs synthesized by using BC extract were used further for investigating the *in vitro* and *in vivo* effect of BC-Fe_3_O_4_ on fungal growth inhibition and plant growth parameters.

### Pathogenicity Assay for *Fusarium oxysporum* f. sp. *lycopersici*

All three tomato varieties indicated the typical symptoms of Fusarium wilt. During a virulence test, characteristic symptoms were noticed after 15–25 days of inoculation. A tomato plant infected with FOL showed a disparity in symptoms on aerial parts and within stem tissues. Initial stages showed the yellowing of lower leaves; however, the drooping of leaves were observed at the later stages of disease (Supplementary Figure 6B). The pith of the infected stem turned brown during severe infection (Supplementary Figure 6C). Correspondingly, lower leaves become withered; eventually, the aerial portion of the plant lost turgidity and wilted down during severe stages of infection. Based on the disease severity index, the highest infection was observed in Rio Grande, followed by Early Boy and Fine Star. No symptoms were observed in uninoculated tomato seedlings (Supplementary Figure 6A). Based on the root-dip inoculation assay-procured culture pathogenic to tomato varieties was identified as *F. oxysporum* f. sp. *lycopersici* (FOL).

### *In vitro* Efficacy of Green-Synthesized Black Coffee–Magnetite Nanoparticles

Recently, magnetic NPs have been gaining great attention due to their efficacy and stability to generate significant development in life sciences and agriculture (Asadi-Kavan et al., 2020). The equilibrium of antimicrobial activity and biocompatibility makes magnetic NPs an attractive candidate for the role of new-generation fungicides (Gudkov et al., 2021).

**FIGURE 11:**
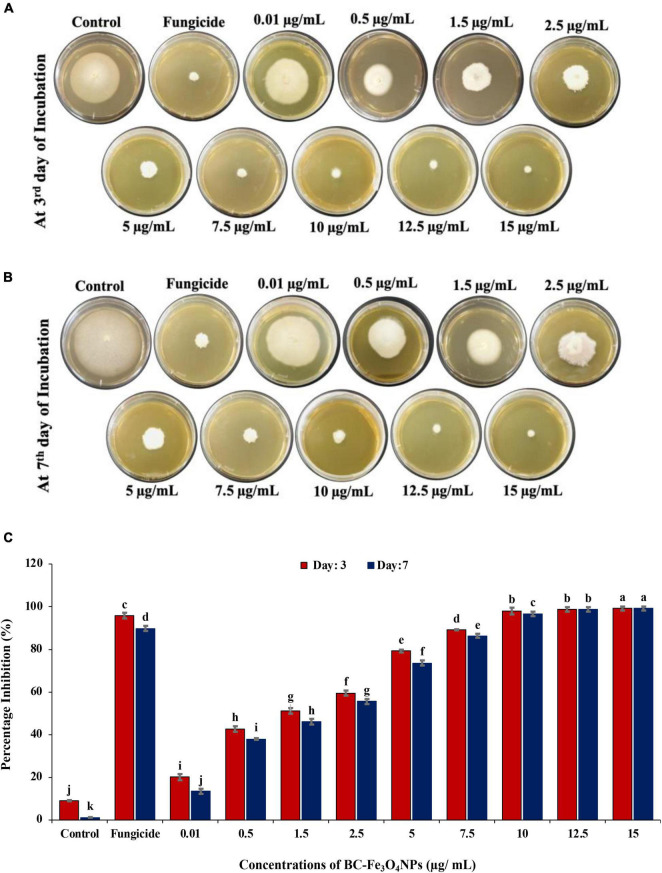
Comparing the effect of BC-Fe_3_O_4_ NPs on mycelial radial growth of *F. oxysporum* at various concentrations (0.01–15 μg/ml) in parallel to control and fungicide treatment after 3 and 7 days of incubation at 28°C. **(A,B)** Plates indicating antifungal activity of BC-Fe_3_O_4_ NPs in PDA plates at third and seventh day. **(C)** Showing percentage inhibition graph of BC-Fe_3_O_4_ NPs on mycelial growth of *F. oxysporum*. Vertical bars represent SD (*n* = 3) between the mean of different replicates of the same treatment. Different letters per column indicate significant differences among treatments, determined by the ANOVA and LSD tests at *P* ≤ 0.05.

Figures 11A–C shows the *in vitro* effect of BC-Fe_3_O_4_ NPs on the mycelial growth of *F. oxysporum* at various concentrations (0.01–15 μg/ml). Figures 11A,B represented the biocidal activity of BC-Fe_3_O_4_ NPs by showing inhibition to fungal mycelial growth in culture plates at the third and seventh day of incubation. The mycelial growth of *F. oxysporum* was significantly (*P* < 0.05) reduced at all concentrations of BC-Fe_3_O_4_ NPs (Figure 11C). As compared to the control, higher concentrations ranged from 10 to 15 μg/ml substantially reduced fungal growth by 96–99%, respectively. Mid-range concentrations (2.5–7.5 μg/ml) also exhibited > 50% inhibition; however, the least activity i.e., < 50 was observed at lowest concentrations (0.01–1.5 μg/ml) both after the third and seventh day of incubation. Similarly, fungicide treatment also expressed significant inhibition. Observations from current data signify that BC-Fe_3_O_4_ NPs at various concentrations instigated significant inhibition in radial growth of the targeted fungus, depending on fungal inoculum and concentrations of NPs (Akpinar et al., 2021).

The antimicrobial properties of NPs are associated with a large surface-area-to-volume ratio and reduced size that efficiently sheaths the microorganism by limiting the oxygen availability required for respiration (Nguyen et al., 2020). The impeding activity of NPs occurs due to metal-ion release, non-oxidative mechanism, and stress induction (Wang et al., 2017). Oxidative stress is triggered by releasing numerous ROS including hydrogen peroxide (H_2_O_2_), singlet oxygen (^1^O_2_), superoxide (O^2–^), and hydroxyl-radical (-OH) (Hasanuzzaman et al., 2020). Earlier reports showed that the formation of ROS regulates the anti-microbial activity of NPs which results in damage of DNA and proteins of microorganisms through oxidative stress (Nguyen et al., 2020). Metal-oxide NPs get absorbed through the cell membrane and deteriorate enzyme activity by interacting with proteins and nucleic acid (Zakharova et al., 2015). As iron is a robust reducing agent, it affects the lipo-polysaccharides and membrane proteins by inducing the disintegration of functional groups. IONPs by Fenton reaction cause the oxidation of intracellular oxygen and lead to the oxidative damage and lysis of cells by penetrating through disrupted membranes (Gaharwar et al., 2017). Thus, it can be stated that green-synthesized BC-Fe_3_O_4_ NPs have the potential to repress plant pathogenic fungi more efficiently as compared to the commercially available fungicides. So, it can also be assumed that the antifungal activity of the synthesized NPs can be correlated with the high dielectric constant of NPs.

### *In vivo* Efficacy of Black Coffee–Magnetite Nanoparticles Under Greenhouse Conditions

**FIGURE 12:**
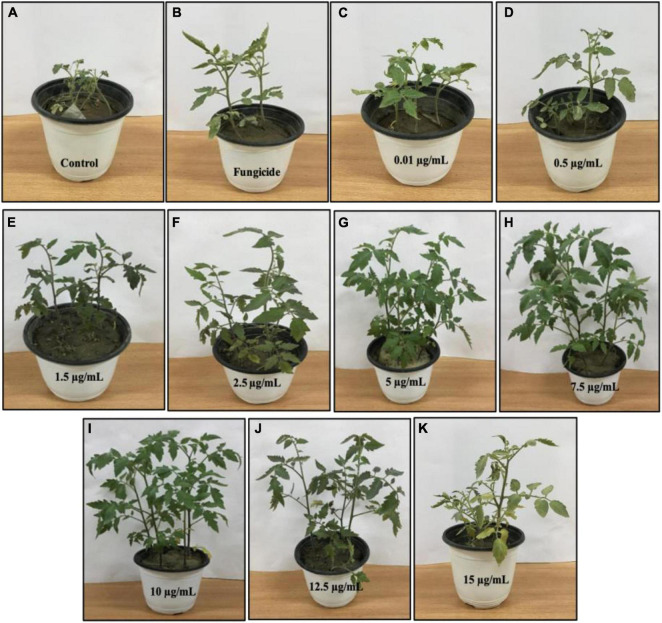
Effect of various concentrations of BC-Fe_3_O_4_ NPs on infected tomato plants under greenhouse pot experiment. **(A)** Pathogen-control, **(B)** non-pathogen control, **(C–K)** different concentrations (0.01, 0.5, 1.5, 2.5, 5, 7.5, 10, 12.5, and 15 μg/ml) of BC-Fe_3_O_4_ NPs.

**FIGURE 13:**
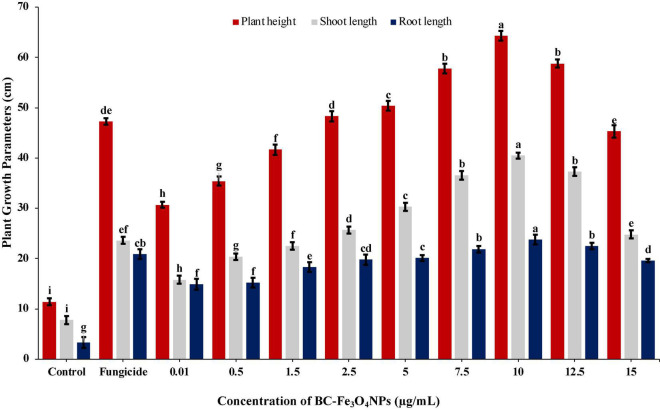
Effect of various concentrations of Fe_3_O_4_ NPs on plant height, root and shoot, and length infected with Fusarium wilt under greenhouse conditions. Vertical bars represent SD (*n* = 3) between the mean of different replicates of the same treatment. Different letters per column indicate significant differences among treatments, determined by ANOVA and LSD tests at *P* ≤ 0.05.

BC-Fe_3_O_4_ NPs were evaluated under greenhouse conditions for exploring their efficacy to suppress the wilt disease of tomatoes caused by FOL. The results of the *in vivo* assay indicated that all concentrations of BC-Fe_3_O_4_ NPs significantly (*P* < 0.05) reduced disease severity and incidence by improving plant growth parameters as compared to the control (Table 3 and Figures 12, 13). The disease-controlling effect of BC-Fe_3_O_4_ NPs on plant growth parameters improved with increased concentrations. Fe_3_O_4_ NPs have the potential to be used for higher productivity and disease management as they can enhance growth parameters in tomato plants with no adverse effect (Abou El-nasr et al., 2015; Rui et al., 2016). In comparison to the control, all treatments showed substantial improvement in plant growth parameters by minimizing disease severity/incidence; however, best results were observed at 10 μg/ml of BC-Fe_3_O_4_ NPs. Plant height and root and shoot length were significantly increased by 64.3, 23.8, and 40.5 cm as compared to the control (11.4, 3.3, and 7.8 cm), respectively (Figure 13). Similarly, fresh and dry weight also indicated substantial improvement (12.8 and 2.27 g) in comparison to the control (1.17 and 0.09 g) treatment (Table 3).

**TABLE 3:** Evaluation of BC-Fe_3_O_4__*NPs*_ against Fusarium wilt of tomato under greenhouse conditions.

Treatments	Disease severity (%)	Disease incidence (%)	Biomass (g)	
			Fresh	Dry
Control	96.8 ± 5.7^a^	100 ± 4.5^a^	1.17 ± 0.1^e^	0.09 ± 0.2^f^
Fungicide	53.3 ± 4.2^g^	58.3 ± 2.2^c^	8.54 ± 0.2^b^	0.57 ± 0.4^d^
0.01 μg/ml-BC-Fe_3_O_4_ NPs	89.3 ± 5.1^b^	94.3 ± 1.4^b^	3.49 ± 0.8^d^	0.23 ± 0.1^e^
0.5 μg/ml-BC-Fe_3_O_4_ NPs	86.5 ± 4.8^c^	90.5 ± 0.9^c^	5.93 ± 0.9^cd^	0.47 ± 0.1^d^
1.5 μg/ml-BC-Fe_3_O_4_ NPs	74.4 ± 4.6^d^	84.7 ± 1.6^d^	7.45 ± 0.3^c^	0.55 ± 0.3^d^
2.5 μg/ml-BC-Fe_3_O_4_ NPs	67.2 ± 7.6^e^	63.8 ± 1.8^e^	8.62 ± 0.7^b^	0.68 ± 0.2^c^
5 μg/ml-BC-Fe_3_O_4_ NPs	56.4 ± 2.5^f^	45.4 ± 1.5^f^	9.17 ± 0.6^ab^	1.02 ± 0.5^b^
7.5 μg/ml-BC-Fe_3_O_4_ NPs	53.8 ± 5.4^g^	43.7 ± 2.4^g^	11.5 ± 0.6^a^	2.21 ± 0.4^a^
10 μg/ml-BC-Fe_3_O_4_ NPs	44.4 ± 6.7^j^	38.6 ± 1.7^j^	12.8 ± 0.5^a^	2.27 ± 0.2^a^
12.5 μg/ml-BC-Fe_3_O_4_ NPs	45.7 ± 5.5^i^	39.3 ± 2.1^i^	10.5 ± 0.4^ab^	1.71 ± 0.1^ab^
15 μg/ml-BC-Fe_3_O_4_ NPs	47.8 ± 7.2^h^	42.8 ± 2.2^h^	8.94 ± 0.8^b^	0.79 ± 0.4^c^

*Different alphabetic letters along the values indicate significant differences determined by the ANOVA and LSD tests at P ≤ 0.05.*

The disease in the control plants was specifically increased and came up to 96.8%; however, the disease severity in tomato plants exposed to BC-Fe_3_O_4_ NPs at concentrations of 10, μg/ml was reduced to 44.4%, respectively. However, with fungicide, a decline of 53.3% in disease severity was observed (Table 3), while the disease incidence in plants exposed to BC-Fe_3_O_4_ NP-treated plants was found to be 38.6% as compared to the 100% control.

The work of Kokina et al. (2020) was in accordance with current findings; plant resistance increased against powdery mildew infection after the application of Fe_3_O_4_ NPs. Lau et al. (2020) stated that Fe_3_O_4_ NPs can act as a resourceful way to deliver active ingredients such as growth-related compounds and fungicides. Additionally, different studies have reported that IONPs increase the rate of seed germination, plant vigor, biomass, yield, and the enhancement of physiological functions (Rui et al., 2016; Hussain et al., 2019; Kasote et al., 2019; Sundaria et al., 2019). Thus, green-synthesized Fe_3_O_4_ NPs not only improved the plant ability to withstand the environmental stresses, but they have also generated positive effects on the growth and development of seedlings by inducing minimum genotoxicity (Shankramma et al., 2016; Plaksenkova et al., 2019). Further, an enhancement in the seed germination of tomato plants with an increase in shoot and root length, after treatment with Fe_2_O_3_ NPs, is very encouraging in the field of agriculture.

### Measurement of Total Phenolics and Defense Enzymes

**FIGURE 14:**
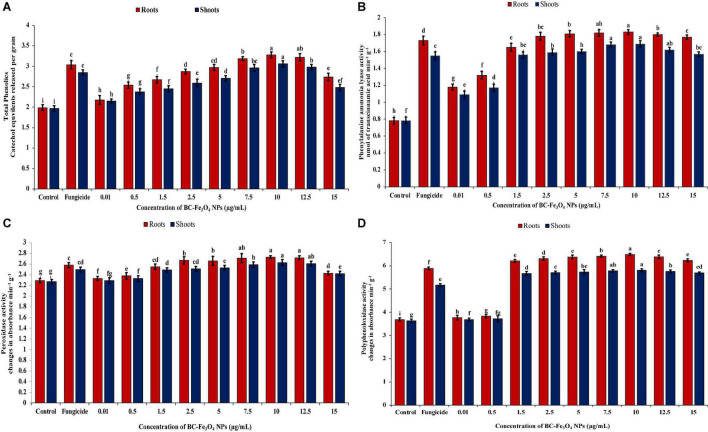
Activities of phenolics and defense enzymes (PAL, POD, and PPO) in roots and shoots of tomato plants treated with different concentrations of BC-Fe_3_O_4_ NPs. **(A)** Phenolic content, **(B)** phenylalanine-ammonia-lyase activity (PAL), **(C)** POD activity, **(D)** polyphenol-oxidase activity (PPO). Vertical bars represent SD (*n* = 3) between the mean of different replicates of the same treatment. Different letters per column indicate significant differences among treatments, determined by the ANOVA and LSD-test at *P* ≤ 0.05.

Infected tomato plants treated with various concentrations of BC-Fe_3_O_4_ NPs (*P* < 0.05) significantly enhanced the quantities of phenolics and other defense-related enzymes (PAL, POD, and PPO) as compared to the control (Supplementary Figure 7). Figure 14A shows the quantities of phenolic content at different concentrations. The highest phenolic activity was detected at 10 μg/ml both in roots and shoots as compared to the control, i.e., 91.9 and 51.8%, respectively. Furthermore, it is indicated that in both cases, i.e., roots and shoots, phenolic activity improved with increasing concentration. However, concentrations of 12.5 and 15 μg/ml showed less phenolic activity, i.e., 67.3 and 50.3% in roots and 50.3 and 25.8% in shoots. In roots, the percentage increases for 0.1–12.5 μg/ml as 9.55, 27.6, 34.2, 44.2, 49.2, and 60.3%, while in shoots, 9.14, 20.8, 24.4, 31.5, 37.5, and 51.3%, respectively, calculated after 45 days of sowing. However, in fungicide treatment, the activity was found to be 52.8 and 44.7% in roots and shoots, respectively. Numerous functions in plants are associated with phenolic activity such as structural stability and protective approach as well as its biocidal activity against several pathogens (Lin et al., 2016). The roots of tomato seedlings treated with different concentrations of BC-Fe_3_O_4_ NPs showed an increased level of phenolic compounds as compared to the control. So, it is confirmed from existing data that disease severity got lowered when phenolic activity was higher in tomato plants after being treated with various concentrations of BC-Fe_3_O_4_ NPs.

Defensive activities in roots and shoots of tomato plants treated with different concentrations of BC-Fe_3_O_4_ NPs were also quantified by enzymatic quantifications. Plants treated with higher doses showed the maximum amount of enzyme activity. Figure 14B showed the activity of PAL enzyme in the roots and shoot of tomato plant treated with various concentrations of BC-Fe_3_O_4_ NPs. For PAL, maximum quantities were measured in plants treated with higher concentrations of BC-Fe_3_O_4_ NPs. An increase of 2.35 and 2.22-folds in roots and shoots at a concentration of 10 μg/ml was observed as compared to the control. Also, an increase of 2.16 and 1.98-folds were observed for roots and shoots in fungicide treatment. Pathogen control revealed the lowest amount of PAL activity. A wavy pattern was observed for PAL activity as shown in decreasing and increasing trends in quantities.

Correspondingly, Figure 14C showed that higher concentrations of BC-Fe_3_O_4_ NPs exhibited maximum activity POD both roots and shoots of the treated plants. Likewise, phenolic and PAL concentrations at 10 μg/ml of BC-Fe_3_O_4_ NPs showed a significant increase of 1.19 and 1.16-folds in contrast to the control and higher doses (12.5 and 15 μg/ml), whereas other concentrations ranging from 0.01 to 7.5 μg/ml showed 1.7–18.3% and 0.9–15% enzyme activity both in roots and shoots, respectively. In comparison to the control, fungicide also exhibited increased POD activity by indicating 1.10-folds in shoots and 1.13-folds in roots, respectively. Similarly, like other defense enzymes, PPO activity was also high in BC-Fe_3_O_4_ NP-treated plants. Relatively higher concentrations exhibited 1.73- and 1.69-folds in roots and 1.58- and 1.56-folds in shoots, while comparing with the control maximum activity for PPO was detected at 10 μg/ml concentration, with a fold increase of 1.76 and 1.60 in roots and shoots, respectively (Figure 14D). Furthermore, it was observed that lower concentrations also revealed increased PPO activity in parallel to the control and fungicide treatment. It is suggested from current findings that BC-Fe_3_O_4_ NPs induced an increased PPO activity in treated plants in comparison to fungicide treatment.

The accumulation of the total phenolic content and antioxidant enzymes were higher in roots in comparison to shoot and equal in fungicide treatment. Plants’ resistance is escorted by amplified enzyme activities tangled in the phenyl-propenoid pathway (Biała and Jasiński, 2018; Yadav et al., 2020). The augmented activities of PAL, PPO, and POD may be concomitant with lignin, melanin, suberin, and quinones synthesis (Araji et al., 2014; Liu et al., 2018; Janusz et al., 2020) that strengthens cell wall and supports the defense barrier against approaching plant pathogens by destroying their pectolytic enzymes (Andersen et al., 2018; Wormit and Usadel, 2018; Köhl et al., 2019). PPO plays a role in limiting disease development by catalyzing phenolic oxidation involved in the initiation of defense resistance against plant diseases (Kaur et al., 2017). Consonant to current findings, earlier literature also reported the increase in secondary metabolites and enzymatic activities after treatment with IONPs in *Moringa oleifera* (Tawfik et al., 2021), *Citrus maxima* (Wang et al., 2017), and *Dracocephalum moldavica* (Moradbeygi et al., 2020), respectively.

### Effect of Black Coffee–Magnetite Nanoparticles on Pathogenesis-Related Protein and Defense Genes

**FIGURE 15:**
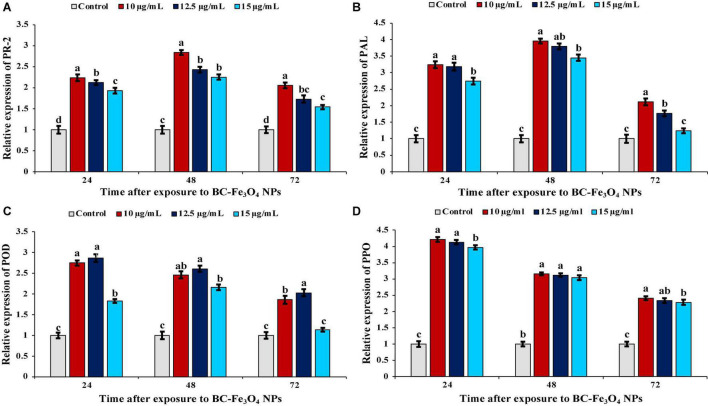
Relative gene expression of pathogenesis-related protein and defense-related genes in tomato plants treated with 10, 12.5, and 15 μg/ml of BC-Fe_3_O_4_ NPs after second foliar spray harvested at 24, 48, and 72 h. **(A)** Expression level of PR-2, **(B)** expression level of PAL, **(C)** expression level of POD, and **(D)** expression level of PPO in tomato roots after treatment with BC-Fe_3_O_4_ NPs. Vertical bars represent SD (*n* = 3) between the mean of different replicates of the same treatment. Different letters per column indicate significant differences among treatments, determined by the ANOVA and LSD test at *P* ≤ 0.05.

Quantitative real-time PCR (qPCR) analysis was performed to quantify the effect of different treatments (10, 12.5, and 15 μg/ml) of BC-Fe_3_O_4_ NPs on transcript expression of pathogenesis-related protein and defense genes in comparison to control inoculated with pathogen only. Fe_3_O_4_ N-treated roots of tomato plants indicated a rapid and significantly high expression of PR-2, PAL, POD, and PPO genes in contrast to control treatment (Figure 15).

In plants, the mechanism for the activation of the defense gene occurs in response to accumulation of stress-mediated metabolites and defense-related enzymes (Chandra et al., 2015). The relative level of PR-2 gene expression significantly increased, following pathogen inoculation at different concentrations of BC-Fe_3_O_4_ NPs (Figure 15A). Maximum expression was recorded after 48 h with a fold increase of 2.84, 2.43, and 2.25 as compared to untreated control, while maximum fold was observed for 10 μg/ml. Pathogenesis-related proteins, PR-2 (β-1,3-glucanase) renowned for antifungal potential, in the plant-induced expression of that gene not only boosts the defense mechanism in plants but also contributes to improving the physiological functions such as cell division, seed maturation, and flower development (Balasubramanian et al., 2012). Thus, the results of the current study indicate that the PR-2 gene was activated early in a substantially higher amount in Fe_3_O_4_ NP-treated roots in comparison to the untreated control. Thus, Fe_3_O_4_ NPs also helped in inducing the expression of PR-proteins that became active in response to pathogen attack. This enzyme directly intricates the hydrolyzing fungal cell-wall glucans (Chandra et al., 2015). Chun and Chandrasekaran (2019) proposed chitosan NPs induced upregulation of PR-proteins and antioxidant genes in tomato plants infected with *F. andiyazi*. In plants, PAL plays a crucial role in the phenylpropanoid pathway and is involved in defense response and secondary metabolism (Chen et al., 2017). The incitement of PAL activity in response to magnetic effect was echoed by the aggregation of phenolic compounds such as anthocyanins and flavonoid that works as a resilient non-enzymatic antioxidant as well as reducing free oxygen radicals in cells (Abdel Latef et al., 2020). As shown in Figure 15B, treatment with BC-Fe_3_O_4_ NPs gradually increased PAL expression, peaked at 48 h at 10 μg/ml of Fe_3_O_4_ NPs; the gene was upregulated by 3.96-folds over untreated control plants. The activation of PAL activity in response to various biotic and abiotic stresses of the environment is supposed to be a part of the plant defense system (Nourozi et al., 2019). Moradbeygi et al. (2020) observed enhanced expression of the PAL gene in Moldavian plants after treatment with IONPs and NaCl. POD is another frequently stated defense-activator enzyme gene that has been reported to be activated in plants under a stress environment (Sharma et al., 2012). A significant transcript level of the POD gene was recorded after 24 h with a fold increase of 2.75, 2.87, and 1.83 in 10, 12.5, and 15 μg/ml of BC-Fe_3_O_4_ NPs (Figure 15C). Increased expression of the POD gene in rice seedlings have been reported after treatment with silver NPs (Gupta et al., 2018). Similarly, the transcript levels of PPO were also upregulated in Fe_3_O_4_ NPs (Figure 15D). The relative expression of PPO in 10 μg/ml treatment was elevated by 4. 21-, 3. 16-, and 2.41-fold at 24, 48, and 72 h, respectively. Expression of genes (PAL, POD, PPO, SOD, and CAT) were highly upregulated in pearl millet infected with downy mildew after treatment with chitosan NPs (Siddaiah et al., 2018). The induced resistance by Fe_3_O_4_ NPs in tomato plants is interrelated with improved enzyme activities and gene expression of PAL, POD, and polyphenol oxidase. A comparison of the current work, i.e., the synthesis and effect of Fe_3_O_4_ NPs on tomato wilt disease, with literature is tabulated in Supplementary Table 1, whereas a comparison of various measures/practices/products that have been used earlier to control this disease, are given in Supplementary Table 2.

## Conclusion

IONPs were green-synthesized using spinach (*S. oleracea*) as starting material. Various extracts such as PJ, WV, PP, BC, AP, and As extracts were used in the synthesis process. FRAP results indicated the strong reducing power of BC extract. The Fe_2_O_3_ phase was observed in NPs synthesized using PJ, WV, PP, AP, and As extracts, whereas the Fe_3_O_4_ phase was observed by using BC extract. Superparamagnetic nature along with high dielectric constant (575) and low tangent loss (0.01) indicated the suitability of Fe_3_O_4_ NPs as an antifungal agent. Spherical NPs with a distinct feature size of ∼20 nm were observed for Fe_3_O_4_ NPs. EDX results showed good elemental composition in green-synthesized NPs using all extracts. XPS confirmed the binding energies of Fe 2p_3/2_ and Fe 2p_1/2_ for IONPs. Raman bands at ∼310 cm^–1^ (T_2__*g*_), ∼550 cm^–1^ (T_2__*g*_), and 670 cm^–1^ (A_1__*g*_) revealed the formation of Fe_3_O_4_ NPs synthesized by using BC extract. Green-synthesized BC-Fe_3_O_4_ NPs showed potent antifungal activity by inhibiting the growth of *F. oxysporum* both during *in vitro* and *in vivo* assays. A significant reduction in disease severity and incidence was observed after treatment with BC-Fe_3_O_4_ NPs. Correspondingly, high defensive biochemical activities were observed for different concentrations of BC-Fe_3_O_4_ NPs, in both the roots and shoots of tomato plants. The activation of PAL activity in response to various biotic and abiotic stresses of the environment is supposed to be a part of the plant defense system. The induced resistance by Fe_3_O_4_ NPs in tomato plants is interrelated with improved enzyme activities and the gene expression of PAL, POD, and polyphenol oxidase. Results demonstrated that Fe_3_O_4_ NPs have the capability to not only control tomato wilt disease but also help to enhance the growth parameters of the tomato plant by strengthening its defense system. All findings suggested that BC-Fe_3_O_4_ NPs were non-phytotoxic and have the potential to become a part of the plant disease management system.

## Data Availability Statement

The original contributions presented in the study are included in the article/Supplementary Material, further inquiries can be directed to the corresponding author/s.

## Author Contributions

HA contributed *in vivo* and *in vitro* study. TB synthesized nanoparticles. TA supervised. AI collected the results. SR supervised along with resources. SN and GL supervised, final checkup, and fundings. All authors contributed to the article and approved the submitted version.

## Conflict of Interest

The authors declare that the research was conducted in the absence of any commercial or financial relationships that could be construed as a potential conflict of interest.

## Publisher’s Note

All claims expressed in this article are solely those of the authors and do not necessarily represent those of their affiliated organizations, or those of the publisher, the editors and the reviewers. Any product that may be evaluated in this article, or claim that may be made by its manufacturer, is not guaranteed or endorsed by the publisher.
