# Supplementary material for: Antifungal Potential of Green Synthesized Magnetite Nanoparticles Black Coffee–Magnetite Nanoparticles Against Wilt Infection by Ameliorating Enzymatic Activity and Gene Expression in Solanum lycopersicum L
Source: Front Microbiol. 2022 Mar 3;13:754292. doi: 10.3389/fmicb.2022.754292 (PMC8928266; doi:10.3389/fmicb.2022.754292)
Supplement: Supplementary file 1 [file Table_1.pdf]

Table S1: Comparison of current work of green synthesized IONPs with literature

Green source/ Plant	Part used / extract	Precursor / Iron salt	Crystal structure / Phase	Shape	Size (nm)	Application	Reference
Uritica dioica	Leaves	$FeCl_3.6H_2O$	Amorphous	spherical	21-71	-	(Ebrahiminezhad et al., 2017)
**Green teabags**	Leaves	$Fe(NO_3)_3.9 H_2O)$	Magnetite (Fe_3O_4)	spherical	25-30	-	(Karade et al., 2017)
**Tannic acid**	-	Iron Powder	Hematite (Fe_2O_3) and Magnetite (Fe_3O_4)	circular	10-30	Antifungal activity	(Parveen et al., 2018)
Lagenaria siceraria	Leaves	$FeCl_3 \cdot 6H_2O$	Fe_3O_4	cube	30-100	Antimicrobial activity	(Kanagasubbulakshmi & Kadirvelu, 2017)
Acacia nilotica	Pods	$FeSO_4 \cdot 7H_2O$	α-Fe_2O_3	Irregular	230	Antibacterial activity	(Da'na et al., 2018)
Platanus orientalis	Leaves	$Fe(NO_3)_3 \cdot 9H_2O$	α-Fe_2O_3 and γ-Fe_2O_3	spherical	~ 38	Antifungal activity	(Devi et al., 2019)
Pinus eldarica	Needle Leaves	$FeCl_3 \cdot 6H_2O$	Amorphous	spherical	8-34	-	(Kheshtzar et al., 2019)
Chaetomorpha antennina	Algal extract	$FeCl_2.4H_2O$	magnetite (Fe_3O_4)	-	8-16 (using XRD)	Nano-fertilizer	(Siji et al., 2017)
Couroupita guianensis	Fruit	$FeCl_3 \cdot 6H_2O$	Magnetite (Fe_3O_4)	spherical	7-80	Antibacterial	(Sathishkumar et al., 2018)
Agrewia optiva & Prunus persica	leaves	$FeCl_2.4H_2O$	Fe_3O_4	Quasi-spherical & spherical	13-70 & 15-60	Antibacterial & Antioxidant	(Mirza et al., 2018)
Spinacia oleracea and Black coffee	***Leaves and Extract***	**-**	***Fe_3O_4***	***spherical***	***~ 20***	***Antifungal (In-vivo, In-vitro)***	***Present work***

References:

Da'na, E., Taha, A., & Afkar, E. (2018). Green Synthesis of Iron Nanoparticles by *Acacia nilotica* Pods Extract and Its Catalytic, Adsorption, and Antibacterial Activities. In *Applied Sciences. 8*, 10. https://doi.org/10.3390/app8101922

Devi, H. S., Boda, M. A., Shah, M. A., Parveen, S., & Wani, A. H. (2019). Green synthesis of iron oxide nanoparticles using *Platanus orientalis* leaf extract for antifungal activity.

Green Processing and Synthesis, *8*(1), 38–45. https://doi.org/10.1515/gps-2017-0145

Ebrahiminezhad, A., Zare-Hoseinabadi, A., Berenjian, A., & Ghasemi, Y. (2017). Green synthesis and characterization of zero-valent iron nanoparticles using stinging nettle (*Urtica dioica*) leaf extract. *Green Processing and Synthesis*, *6*(5), 469–475. https://doi.org/doi:10.1515/gps-2016-0133

Kanagasubbulakshmi, S., & Kadirvelu, K. (2017). Green synthesis of Iron oxide nanoparticles using *Lagenaria siceraria* and evaluation of its antimicrobial activity. *Life Science Journal*, *2*, 422–427.

Karade, V. C., Waifalkar, P. P., Dongle, T. D., Sahoo, S. C., Kollu, P., Patil, P. S., & Patil, P. B. (2017). Greener synthesis of magnetite nanoparticles using green tea extract and their magnetic properties. *Materials Research Express*, *4*(9), 96102. https://doi.org/10.1088/2053-1591/aa892f

Kheshtzar, R., Berenjian, A., Taghizadeh, S.-M., Ghasemi, Y., Asad, A. G., & Ebrahiminezhad, A. (2019). Optimization of reaction parameters for the green synthesis of zero valent iron nanoparticles using pine tree needles. *Green Processing and Synthesis*, *8*(1), 846–855. https://doi.org/doi:10.1515/gps-2019-0055

Mirza, A. U., Kareem, A., Nami, S. A. A., Khan, M. S., Rehman, S., Bhat, S. A., Mohammad, A., & Nishat, N. (2018). Biogenic synthesis of iron oxide nanoparticles using *Agrewia optiva* and *Prunus persica* phyto species: Characterization, antibacterial and antioxidant activity. *Journal of Photochemistry and Photobiology B: Biology*, *185*, 262–274. https://doi.org/https://doi.org/10.1016/j.jphotobiol.2018.06.009

Parveen, S., Wani, A. H., Shah, M. A., Devi, H. S., Bhat, M. Y., & Koka, J. A. (2018). Preparation, characterization and antifungal activity of iron oxide nanoparticles. *Microbial Pathogenesis*, *115*, 287–292. https://doi.org/https://doi.org/10.1016/j.micpath.2017.12.068

Sathishkumar, G., Logeshwaran, V., Sarathbabu, S., Jha, P. K., Jeyaraj, M., Rajkuberan, C., Senthilkumar, N., & Sivaramakrishnan, S. (2018). Green synthesis of magnetic Fe_3O_4 nanoparticles using *Couroupita guianensis* Aubl. fruit extract for their antibacterial and cytotoxicity activities. *Artificial Cells, Nanomedicine, and Biotechnology*, *46*(3), 589–598. https://doi.org/10.1080/21691401.2017.1332635

Siji, S., Njana, J., Amrita, P. J., Raj, A., Vishnudasan, D., & Manoj, P. K. (2017). Green synthesized iron nanoparticles and its uptake in *pennisetum glaucum* — A nanonutriomics approach. *2017 International Conference on Technological Advancements in Power and Energy (TAP Energy)*, 1–8. https://doi.org/10.1109/TAPENERGY.2017.8397338
