# Supplementary material for: Antifungal Potential of Green Synthesized Magnetite Nanoparticles Black Coffee–Magnetite Nanoparticles Against Wilt Infection by Ameliorating Enzymatic Activity and Gene Expression in Solanum lycopersicum L
Source: Front Microbiol. 2022 Mar 3;13:754292. doi: 10.3389/fmicb.2022.754292 (PMC8928266; doi:10.3389/fmicb.2022.754292)
Supplement: Supplementary file 2 [file Table_2.DOCX]

**Table S2: Comparison between different ways of treatments (fungicides, biological control agents and nanoparticles) for management of Fusarium wilt disease of Tomato**

| **Chemical Fungicides and Formulations** | **Products** | **Chemical group/ micro-organism**  **/salt/source** | **Mode of action** | **Disease Reduction** | **Effects** | **Reference** |
| --- | --- | --- | --- | --- | --- | --- |
|  | Nativo 75 % | Tebuconazole + Trifloxystrobin | Inhibit spore germination | 30.14% | Fungicide residues left on tomato fruit, Phototoxic to tomato seedlings  CYP inhibition, Biotransformation, Neurotoxicity, Human health risk | (Anitha & Rabeeth, 2009; Amini & Dzhalilov, 2010; Charoenporn et al., 2010; Yang et al., 2011; Lozowicka et al., 2015; Akhtar et al., 2017; Abd-Elhaleem, 2020) |
|  | Fludioxonil (Maxim SC) | Phenylpyrrole | Inhibit hyphae formation by effecting signal transduction pathway | 69% |  |  |
|  | Prochloraz 50 % WP | Imidazole | Inhibit conidial development | 22% |  |  |
|  | Carbendazim | Benzimidazole | Inhibit mitosis in fungi | 19% |  |  |
| **Biological Control Agents** | Plant growth Promoting Rhizobacteria (PGPR) | *(Pseudomonas fluorescence* & Streptomyces sp. | Induced systemic resistance | 41.6% | Intolerant to environmental stress i.e. heat, UV-radiations, limited shelf life, required effective selection and screening procedures | (Amini & Dzhalilov, 2010; Boukerma et al., 2016; Vejan et al., 2016; Cotes et al., 2018; Mohammed et al., 2019; Hussein & Al-Dulaimi, 2020) |
|  |  | *Pseudomonas fluorescence* & *Bacillus subtilis* | Production of ammonia, indole acetic acid (IAA), phosphate solubilization | ND |  |  |
|  |  | TEAGRO | Antagonistic effect | 26.06% |  |  |
|  |  | *Pseudomonas fluorescens* PF15  & *Pseudomonas putida* PP27 | Induced Systemic Resistance | 32-72% |  |  |
|  | Fungus based | TRICOTEC^®^  richoderma koningiopsis  Th003  richoderma koningiopsis  Th003  Trichoderma koningiopsis  Th003  Trichoderma koningiopsis  Th003  Trichoderma koningiopsis  Th003  Trichoderma koningiopsis  Th003  (*Trichoderma koningiopsis* Th003) | Extensive root colonization by Th003 | 42% |  |  |
| **Nanoparticles** | Silver (Ag) NPs | Silver nitrate | ROS generation in fungal cells and enhancement of defense mechanism in host plant | 31-100% | Stimulated plant growth, reduce cost of production, balanced nutrient supply, developing plant resistance, biodegradability, adaptive to climate change, expressing stress gene | (Ahmed et al., 2016; Ashraf et al., 2020; Elmer & White, 2016; Sathiyabama & Charles, 2015; Shang et al., 2019) |
|  | Copper oxide NPs  (Graphene-oxide nanosheets) | Natural graphite powder, cupric chloride | Induces cell death by forming pores and pits on fungal cell wall | 30% |  |  |
|  | Nano-cerium (CeO_2_) | University of California, Center for Environmental Implications of Nanotechnology (UC CEIN). | ROS generation | 53% |  |  |
|  | Nickel (Ni) NPs | Cheorwon Plasma Research Institute, Cheorwon-gun, Gangwon-do, Korea | Overproduction of extra-cellular enzymes by fungus | >50% |  |  |
|  | Metallic oxide NPs (CuO, MnO & ZnO) | US Research Nanomaterials (Houston, TX) | Nanoparticles of micronutrients boost host defense mechanism | 28-31% |  |  |
|  | Chitosan NPs  (Fungal-cell wall polymer) | Fol cell wall polymer | Upregulation of defense related genes | 81% |  |  |
|  | Fe_3_O_4_ NPs | Spinach + various extracts | Results demonstrated that Fe_3_O_4_ NPs have capability to not only control tomato wilt disease but these NPs can also be helpful in enhancing the growth parameters of tomato plant by strengthening its defense system. | 95-100% | Stimulated plant growth, Reduced production and application cost of developing plant resistance, Strengthened defense system of plant and are non-toxic | ***Current Study*** |

**References:**

Abd-Elhaleem, Z. (2020). Pesticide residues in tomato and tomato products marketed in Majmaah province, KSA, and their impact on human health. *Environmental Science and Pollution Research*, *27*. https://doi.org/10.1007/s11356-019-07573-x

Ahmed, A. I. S., Yadav, D. R., & Lee, Y. S. (2016). Applications of Nickel Nanoparticles for Control of Fusarium Wilt on Lettuce and Tomato. *International Journal of Innovative Research in Science, Engineering and Technology,5* (5), 7378-7385. https://doi.org/10.15680/IJIRSET.2016.0505132

Akhtar, T., Shakeel, Q., Sarwar, G., Muhammad, S., Iftikhar, Y., Ullah, Mubeen, M., & Hannan, A. (2017). Evaluation of fungicides and biopesticides for the control of Fusarium wilt of tomato. *Pakistan Journal of Botany*, *28*, 1–18.

Amini, J., & Dzhalilov, F. (2010). The Effects of Fungicides on *Fusarium Oxysporum* f.sp. *Lycopersici* Associated with Fusarium Wilt of Tomato. *Journal of Plant Protection Research*, *50*. https://doi.org/10.2478/v10045-010-0029-x

Anitha, A., & Rabeeth, M. (2009). Control of Fusarium Wilt of Tomato by Bioformulation of Streptomyces griseus in Green House Condition. *African Journal of Biotechnology*, *1*(2), 9-14.

Ashraf, H., Anjum, T., Riaz, S., & Naseem, S. (2020). Microwave-Assisted Green Synthesis and Characterization of Silver Nanoparticles Using Melia azedarach for the Management of Fusarium Wilt in Tomato. *Frontiers in Microbiology*, *11*, 238. https://doi.org/10.3389/fmicb.2020.00238

Boukerma, L., Messaoud, B., Charif, A., & Khelifi, L. (2016). Activity of plant growth promoting rhizobacteria (PGPRs) in the biocontrol of tomato Fusarium wilt. *Plant Protection Science*, *53*. https://doi.org/10.17221/178/2015-PPS

Charoenporn, C., Kanokmedhakul, S., Lin, F., Poeaim, S., & Soytong, K. (2010). Evaluation of bio-agent formulations to control Fusarium wilt of tomato. *African Journal of Biotechnology*, *9*.

Cotes, A., Moreno, C., Correal, C., Villamizar, L., & Gómez Alvarez, M. (2018). Biological control of tomato Fusarium wilt and whiteflies with two fungal biopesticides. *Acta Horticulturae*, 129–138. https://doi.org/10.17660/ActaHortic.2018.1207.17

Elmer, W. H., & White, J. C. (2016). The use of metallic oxide nanoparticles to enhance growth of tomatoes and eggplants in disease infested soil or soilless medium. *Environmental Science: Nano*, *3*(5), 1072–1079. https://doi.org/10.1039/C6EN00146G

Hussein, H. Z., & Al-Dulaimi, S. I. (2020). Biological management of fusarium wilt on tomato caused by *Fusarium oxysporum* f. sp. *lycospersici* by some plant growth-promoting bacteria. *BioRxiv*, 2020.08.21.262212. https://doi.org/10.1101/2020.08.21.262212

Lozowicka, B., Abzeitova, E., Sagitov, A., Kaczyński, P., Toleubayev, K., & Li, A. (2015). Studies of pesticide residues in tomatoes and cucumbers from Kazakhstan and the associated health risks. *Environmental Monitoring and Assessment*, *187*, 4818. https://doi.org/10.1007/s10661-015-4818-6

Mohammed, B. L., hussein, R. A., & Toama, F. N. (2019). Biological control of Fusarium wilt in tomato by endophytic rhizobactria. *Energy Procedia*, *157*, 171–179. https://doi.org/https://doi.org/10.1016/j.egypro.2018.11.178

Sathiyabama, M., & Charles, R. E. (2015). Fungal cell wall polymer-based nanoparticles in protection of tomato plants from wilt disease caused by Fusarium oxysporum f.sp. lycopersici. *Carbohydrate Polymers*, *133*, 400–407. https://doi.org/https://doi.org/10.1016/j.carbpol.2015.07.066

Shang, Y., Hasan, M. K., Ahammed, G. J., Li, M., Yin, H., & Zhou, J. (2019). Applications of Nanotechnology in Plant Growth and Crop Protection: A Review. *Molecules (Basel, Switzerland)*, *24*(14), 2558. https://doi.org/10.3390/molecules24142558

Vejan, P., Abdullah, R., Khadiran, T., Ismail, S., & Nasrulhaq Boyce, A. (2016). Role of Plant Growth Promoting Rhizobacteria in Agricultural Sustainability-A Review. *Molecules (Basel, Switzerland)*, *21*(5), 573. https://doi.org/10.3390/molecules21050573

Yang, C., Hamel, C., Vujanovic, V., & Gan, Y. (2011). Fungicide: Modes of Action and Possible Impact on Nontarget Microorganisms. *International Scholarly Research Notices Ecology*, *2011*. https://doi.org/10.5402/2011/130289
