# Supplementary material for: Antifungal Potential of Green Synthesized Magnetite Nanoparticles Black Coffee–Magnetite Nanoparticles Against Wilt Infection by Ameliorating Enzymatic Activity and Gene Expression in Solanum lycopersicum L
Source: Front Microbiol. 2022 Mar 3;13:754292. doi: 10.3389/fmicb.2022.754292 (PMC8928266; doi:10.3389/fmicb.2022.754292)
Supplement: Supplementary file 3 [file Data_Sheet_1.DOCX]

**Figure S1: Graphs showing (a) FRAP and (b) total polyphenols in spinach and various extracts (PJ, WV, PP, BC, AP and As) used for the synthesis of iron oxide nanoparticles (IONPs).**





**Figure S2: Crystallite size and dislocation density of Iron oxide nanoparticles synthesized by (a) PJ, (b) WV, (c) PP, (d) BC, (e) AP and (f) As extracts**







**Figure S3: Variation in (a) saturation magnetization (M_s_) and (b) coercivity (H_c_) of green synthesized IONPs**


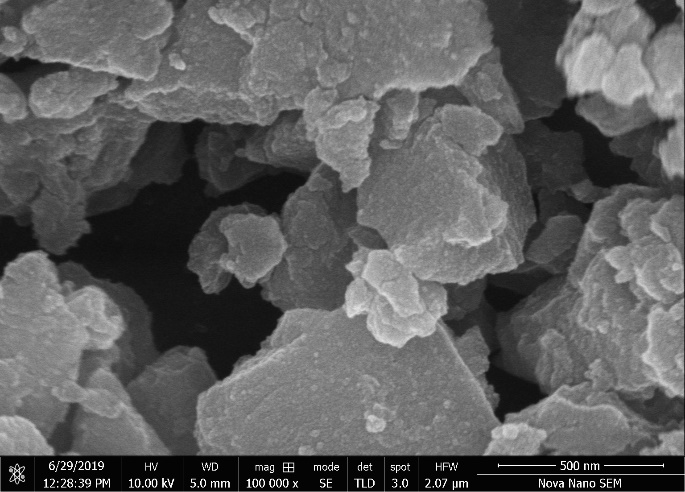

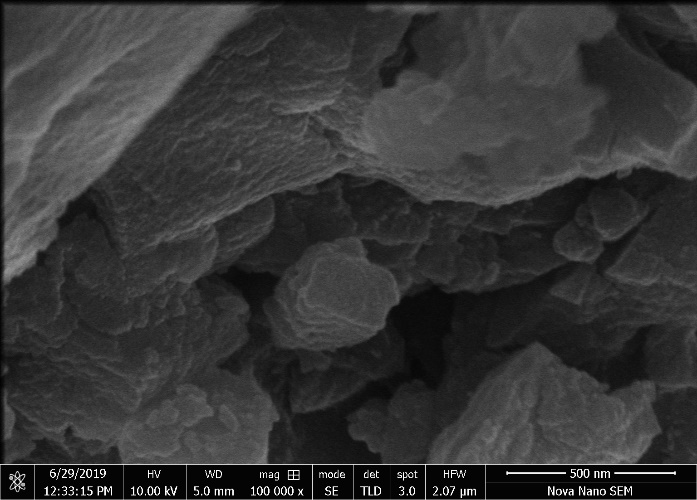

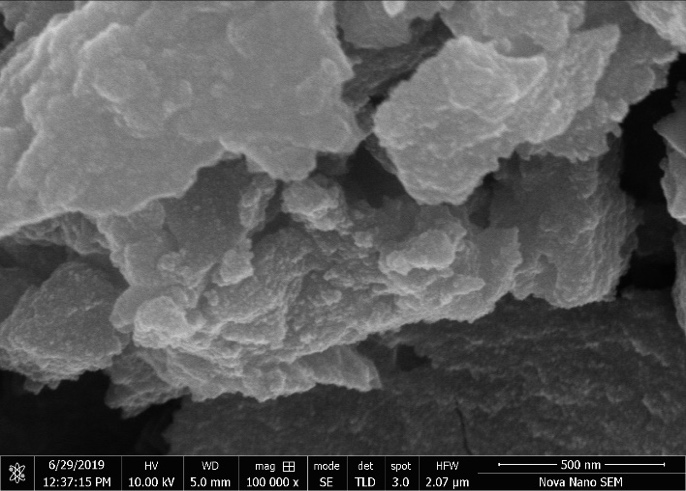

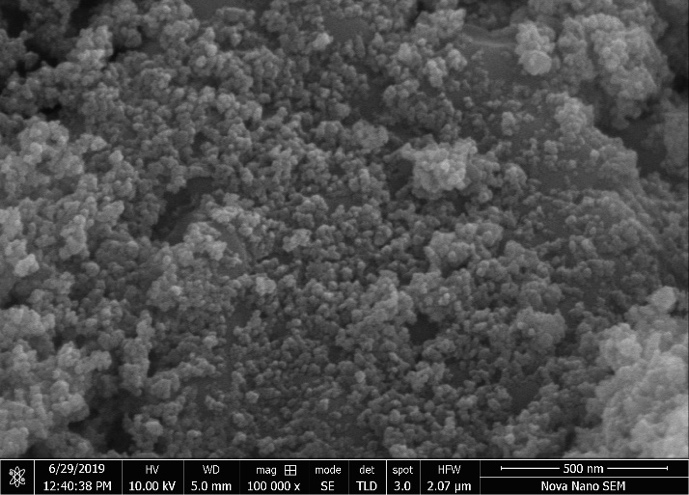

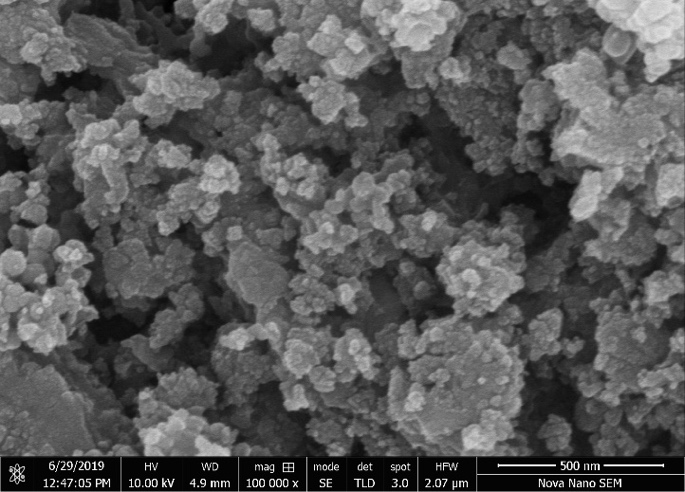


**(a)**

**(c)**

**(b)**

**(d)**

**(e)**

**Figure S4: SEM images of green synthesized IONPs using (a) PJ, (b) WV, (c) PP, (d) AP and (e) As extracts (magnification 500 nm).**


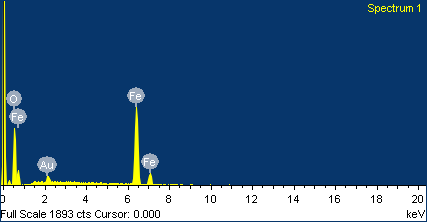


**(a)**

| Element | Weight% | Atomic% |  |
| --- | --- | --- | --- |
| O K | 15.34 | 39.55 |  |
| Fe K | 80.75 | 59.63 |  |
| Au M | 3.90 | 0.82 |  |
| Totals | 100.00 |  |  |


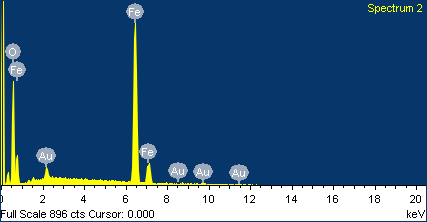


| Element | Weight% | Atomic% |  |
| --- | --- | --- | --- |
| O K | 13.98 | 36.87 |  |
| Fe K | 82.56 | 62.39 |  |
| Au M | 3.46 | 0.74 |  |
| Totals | 100.00 |  |  |

**(b)**


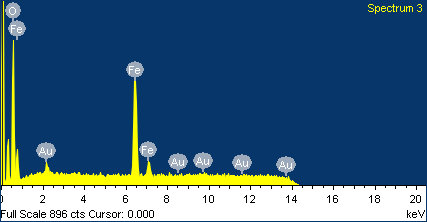


| Element | Weight% | Atomic% |  |
| --- | --- | --- | --- |
| O K | 23.26 | 52.32 |  |
| Fe K | 72.92 | 46.99 |  |
| Au M | 3.82 | 0.70 |  |
| Totals | 100.00 |  |  |

**(c)**


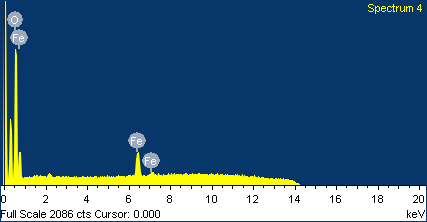


| Element | Weight% | Atomic% |  |
| --- | --- | --- | --- |
| O K | 50.43 | 78.03 |  |
| Fe K | 49.57 | 21.97 |  |
| Totals | 100.00 |  |  |

**(d)**


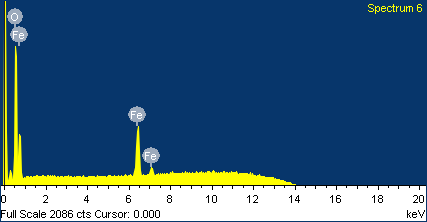


| Element | Weight% | Atomic% |  |
| --- | --- | --- | --- |
| O K | 37.11 | 67.31 |  |
| Fe K | 62.89 | 32.69 |  |
| Totals | 100.00 |  |  |

**(e)**

**Figure S5: EDX spectra of IONPs synthesized using (a) PJ, (b) WV, (c) PP, (d) AP and (e) As extracts**


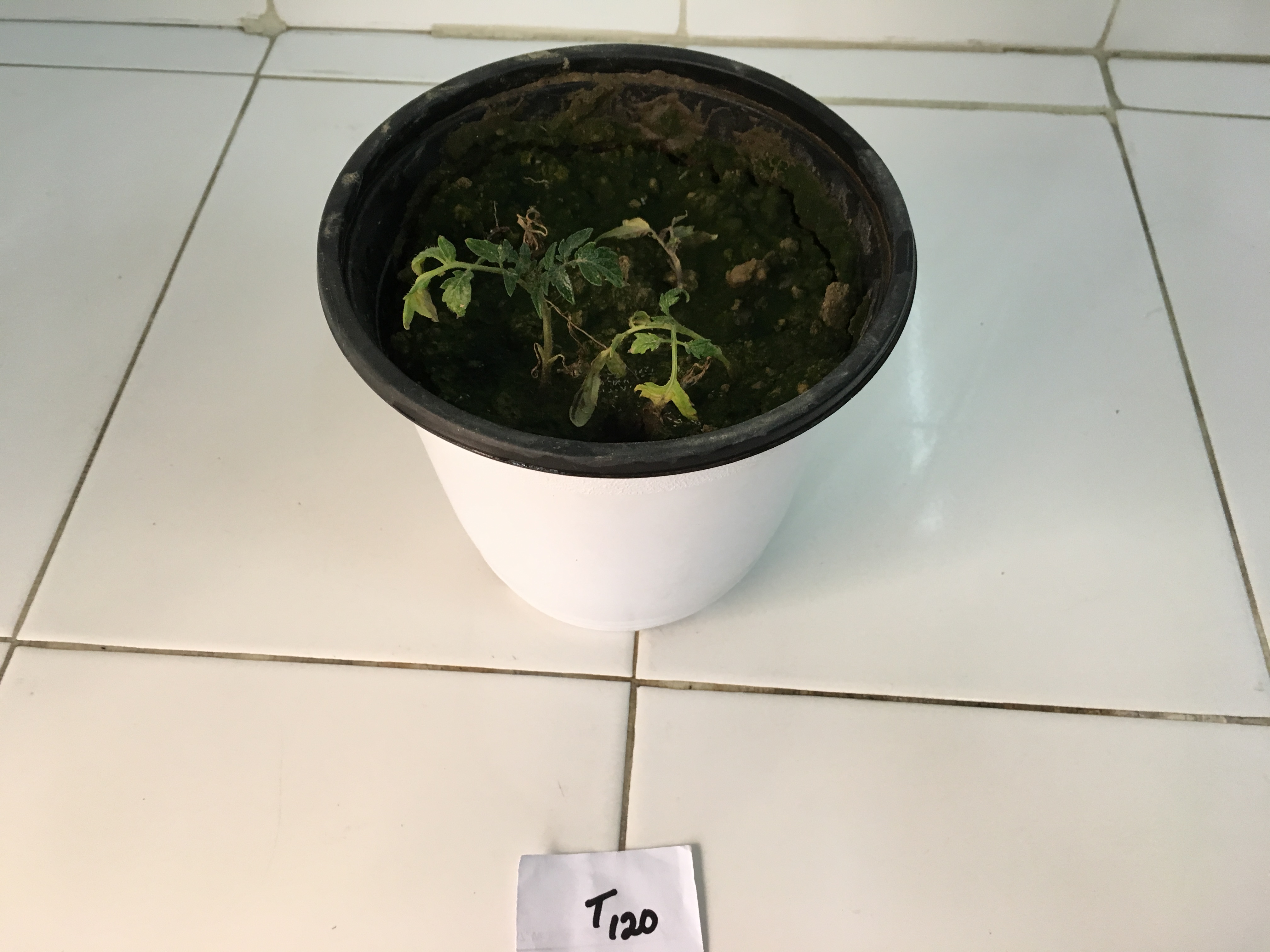

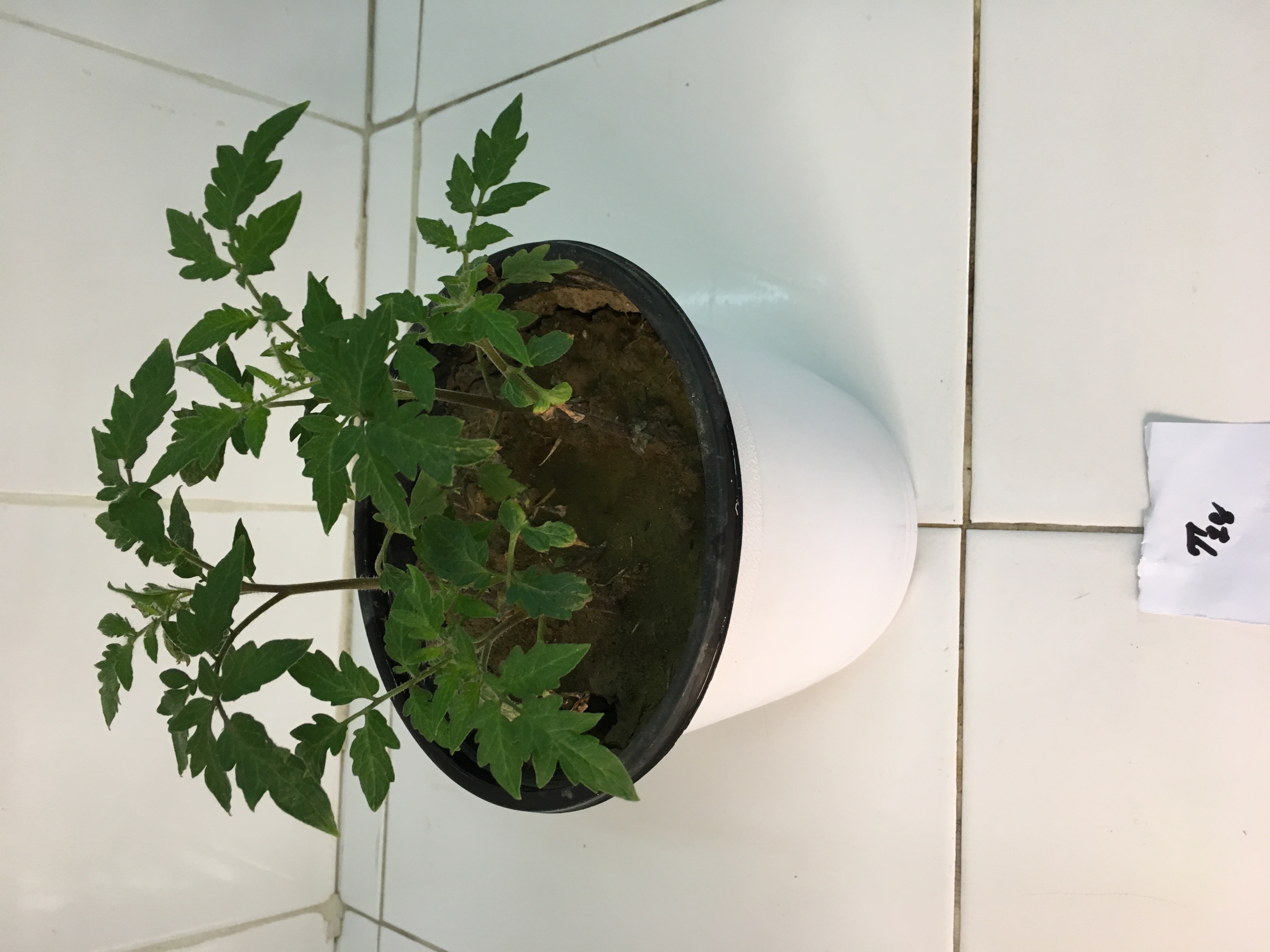

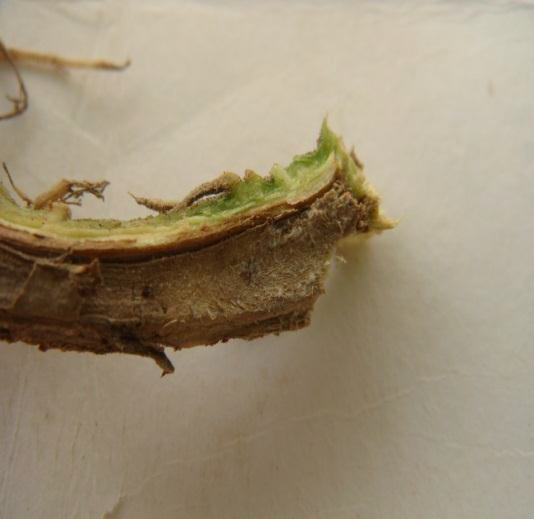


**(a)**

**(b)**

**(c)**

**Figure S6: Pathogenicity assay for *F. oxysporum* f.sp. *lycopersici* to cause wilt infection in tomato plants to prove Koch’s postulates. (a): Healthy tomato plant; (b): Infected plants showing wilt typical symptoms; (c) Stem of infected plant indicating browning of tissues.**


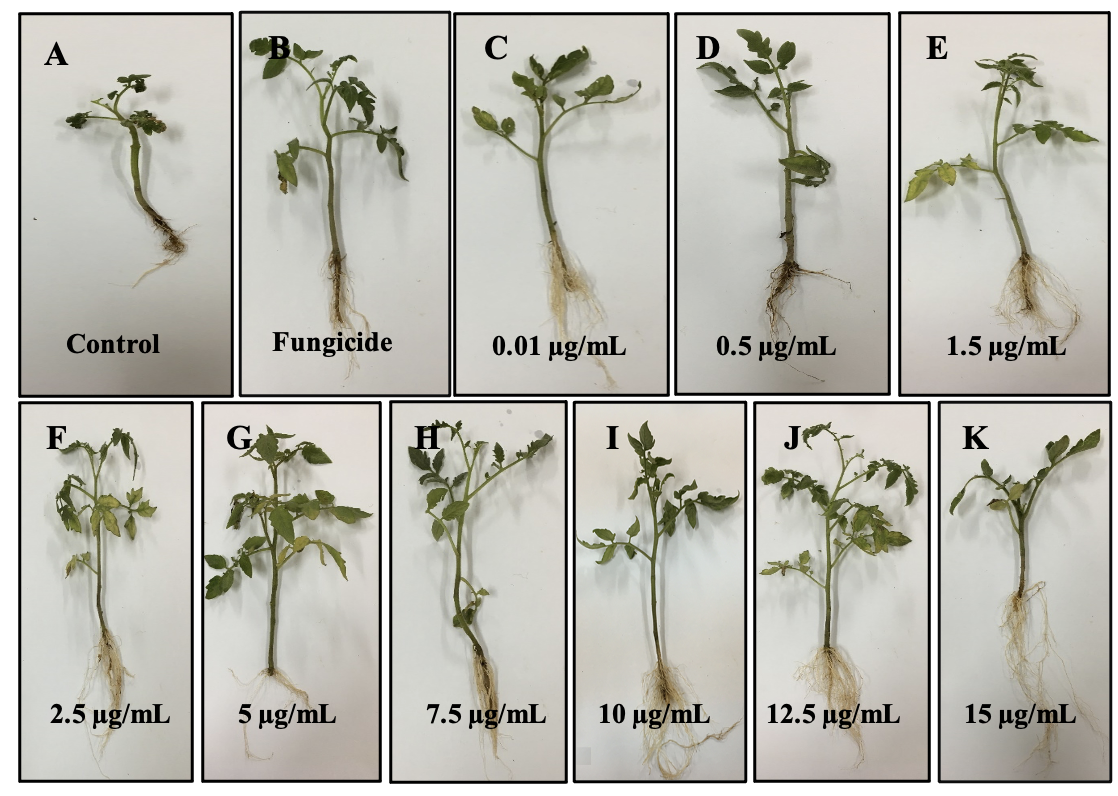


**Figure S7: Effect of various concentrations of BC-Fe_3_O_4_ NPs on root and shoot length of tomato plants infected with F. oxysporum under greenhouse conditions. A: Control, B: Fungicide, C-K: Different concentrations (0.01 µg/mL, 0.5 µg/mL, 1.5 µg/mL, 2.5 µg/mL, 5 µg/mL, 7.5 µg/mL, 10 µg/mL, 12.5 µg/mL and 15 µg/mL) of BC-Fe_3_O_4_ NPs**
